# Reward enhances resilience to chronic social defeat stress in mice: Neural ECs and mGluR5 mechanism *via* neuroprotection in VTA and DRN

**DOI:** 10.3389/fpsyt.2023.1084367

**Published:** 2023-02-16

**Authors:** Peixia Shi, Linlin Hu, Hui Ren, Qin Dai

**Affiliations:** ^1^Department of Medical Psychology, Army Medical University, Chongqing, China; ^2^Department of Neurology, Xinqiao Hospital, Army Medical University, Chongqing, China; ^3^Department of Nursing Psychology, Army Medical University, Chongqing, China

**Keywords:** reward, stress resilience, social defeat stress, ECs, mGluR5

## Abstract

**Introduction:**

Stress often leads to emotional disorders such as depression. The reward might render this effect through the enhancement of stress resilience. However, the effect of reward on stress resilience under different intensities of stress needs more evidence, and its potential neural mechanism has been poorly revealed. It has been reported that the endogenous cannabinoid system (ECs) and downstream metabolic glutamate receptor 5 (mGluR5) are closely related to stress and reward, which might be the potential cerebral mechanism between reward and stress resilience, but there is a lack of direct evidence. This study aims to observe the effect of reward on stress resilience under different intensities of stress and further explore potential cerebral mechanisms underlying this effect.

**Methods:**

Using the chronic social defeat stress model, we applied reward (accompanied by a female mouse) under different intensities of stress in mice during the modeling process. The impact of reward on stress resilience and the potential cerebral mechanism were observed after modeling through behavioral tests and biomolecules.

**Results:**

The results showed that stronger stress led to higher degrees of depression-like behavior. Reward reduced depression-like behavior and enhanced stress resilience (all *p*-value <0.05) (more social interaction in the social test, less immobility time in the forced swimming test, etc.), with a stronger effect under the large stress. Furthermore, the mRNA expression levels of CB1 and mGluR5, the protein expression level of mGluR5, and the expression level of 2-AG (2-arachidonoylglycerol) in both ventral tegmental area (VTA) and dorsal raphe nucleus (DRN) were significantly upregulated by reward after modeling (all *p*-value <0.05). However, the protein expression of CB1 in VTA and DRN and the expression of AEA (anandamide) in VTA did not differ significantly between groups. Intraperitoneal injection of a CB1 agonist (URB-597) during social defeat stress significantly reduced depression-like behavior compared with a CB1 inhibitor (AM251) (all *p*-value <0.05). Interestingly, in DRN, the expression of AEA in the stress group was lower than that of the control group, with or without reward (all *p*-value <0.05).

**Discussion:**

These findings demonstrate that combined social and sexual reward has a positive effect on stress resilience during chronic social defeat stress, potentially by influencing the ECs and mGluR5 in VTA and DRN.

## 1. Introduction

Stress is often a risk factor for mental disorders such as depression (1). Experiencing stress is 2.5 times more in depressed patients compared to healthy controls and those in community samples, 80% of depressive episodes are preceded by major life events (2, 3). Individuals might confront a variety of stressors, i.e., acute stress or chronic stress (4, 5); however, the effect of different intensities of stress on depression remains unclear.

Notably, stress would not affect everyone similarly, i.e., individuals susceptible to stress may poorly cope with stressors and exhibit inadaptable responses that might result in a persistent stressful status, while individuals resilient to stress may view adversity as less threatening and exhibit adaptive physiological and psychological responses (6). Therefore, finding ways to improve resilience to stress might provide a new idea to prevent and cure depression. Resilience can be defined as the ability to overcome adversity and stress or to achieve a relatively good outcome despite risky experiences (7). Stress resilience is a more specific concept, which is defined as a successful adaptation to stressors without developing significant psychopathology (8). It has been reported that stress resilience is influenced by genetic (9), developmental (10), cognitive (11), psychological (12), and neurobiological (13–15) factors. Studies further showed that the protective effect of stress resilience on the negative outcomes of stress (15) was mediated by environmental factors (16), neural circuits (17), neurotransmitters, and molecular pathways (15, 18, 19). However, how to improve resilience to stress effectively needs further exploration.

The reward is an important part of life, which had different degrees (20). The primary reward represents those immediately influence survival, such as food and reproduction. In contrast, the secondary reward represents those that may not directly impact survival but facilitate these survival behaviors, including money and positive social experiences (21, 22). Reward produces pleasure, positive behavior, and direct learning (20) and has a positive effect on stress-related mental disorders (23). Some studies have investigated the relationship between reward and stress-related disorders, which found that high reward experience was associated with reduced affective symptoms after previous exposure to childhood adversity or recent stressful life events (24). Participants with high sensitivity to reward reported a stronger positive effect following stress, in which a robust reward system serves as a protective mechanism against stress-related adverse outcomes (25). Further proof in animal experiments showed that natural rewards, such as palatable food, provided a general stress reduction, potentially *via* structural and functional plasticity (26). The evidence showed that adequate consumption of sweetened drink attenuated hypothalamic-pituitary-adrenocortical axis stress responses (27) while providing reward at fixed intervals altered fear-avoidant behavior, albeit modestly, in zebrafish (28). Social reward, as a kind of important secondary reward for both animals and human beings, represents close social connections and positive interactions (29), which also indicated an effect in rendering the negative effect of stress (26). These results suggested the possibility of using rewards as an effective way to enhance stress resilience. However, although previous studies have discussed the changes in reward response to stress (including physiological changes), most of the reward was natural, given transitorily or before stress (26–28), while the social reward experience corresponding to stress resilience during chronic stress has not been reported. In this study, we intended to validate the reward system changes under chronic social defeat stress and combined social and sexual reward manner by using the activation status of dopamine receptors (D1 and D2) as well as by the protein expression levels of tyrosine hydroxylase (an enzyme involved in the synthesis of catecholamine neurotransmitters dopamine). Moreover, stress has different intensities, and it remains unclear whether the effect of reward is the same or different under different intensity levels of stress.

Increasing evidence revealed an essential role of neural reward circuitry in the pathophysiology and symptomatology of a range of mood disorders, such as depression (30). Here, we focused on the ventral tegmental area (VTA) and dorsal raphe nucleus (DRN) for their involvement in both reward and stress-related mental disorders. The study has shown that the VTA, a brain region that is most closely related to reward, determines the susceptibility and resilience to social defeat stress, with susceptibility phenotype exhibiting depression-like behavior (31). Furthermore, studies reported that phasic activation of VTA neurons projecting to the nucleus accumbens (NAc), but not to the medial prefrontal cortex (mPFC), induced susceptibility to social defeat stress. Conversely, optogenetic inhibition of the VTA-NAc projection increased resilience, whereas inhibition of the VTA-mPFC projection promoted susceptibility (32). Similarly, 5-HT neurons in the dorsal raphe nucleus (DRN) were also closely related to stress and reward. DRN serotonin transporter-deficient mice exhibited extensive depression-like phenotype, such as anhedonia, learned helplessness, and social avoidance (33). Meantime, the study confirmed that DRN 5-HT neurons positively encoded a wide range of reward signals during anticipatory and consummatory phases of reward, such as sucrose, food, sex, and social interaction (34). Optogenetic activation of DRN Pet-1 neurons (90% 5-HT neurons) produced strong reward experience, reinforcement in behavior, and efficient learning (35). The results confirmed that both VTA and DRN are closely related to stress and reward; however, whether they were crucial brain mechanisms where reward affects resilience to stress and potential underlying molecular mechanism need to be further explored.

The endogenous cannabinoid system (ECs) has shown a neuroprotection function (36–39). In brief, it consists of cannabinoid receptors (CBRs, namely, CB1R and CB2R) and endogenous ligands (eCBs), including the most studied 2-arachidonoylglycerol (2-AG) and anandamide (AEA). The study found that ECs regulated reward-seeking behavior by modulating dopamine signals in the VTA region (40). Disrupting endocannabinoid signaling dramatically reduced reward-seeking behavior, whereas augmenting levels of the endocannabinoid 2-AG increased cue-evoked dopamine concentrations and reward-seeking behavior (41). Meantime, studies suggested that stress altered endocannabinoid transmission in the brain. Specifically, stress elicited the rapid formation of endocannabinoids in the periaqueductal gray matter of the midbrain (42) and altered endocannabinoid content in the limbic forebrain, amygdala, striatum, and prefrontal cortex (43, 44). The results suggested that ECs play a vital role in both stress and reward processes, which might be a crucial molecular mechanism underlying the effect of reward on resilience to stress.

Moreover, the metabotropic glutamate receptor 5 (mGluR5) is a Gαq/11-coupled receptor, which has been mainly found at the postsynaptic site, acting together with the CB1 receptor to promote neuroprotection (45, 46). Some studies pointed out the essential role of mGluR5 in promoting stress resilience and suggested that a deficiency in mGluR5-mediated signaling in the NAc might represent an endophenotype for stress-induced depression (47). A previous study showed that mGluR5 protein expression and bonding levels were reduced in the hippocampus of postmortem depression patients (48). Another study reported that chronic mild stress (CMS) upregulated mGluR5 protein expression in CA1 but downregulated mGluR5 in the CA3 region of the rat hippocampus (49). Exposed to various stressful stimuli, mGluR5–/– mice showed more depression-like behaviors than the control mice (47, 50). The connection between mGluR5 and reward could be further manifested in drug abuse studies. The mGluR5 mediated behavioral responses toward cocaine and also participated in the reward effect of regulating alcohol (51). The results confirmed that mGluR5 is closely related to reward and stress, which might be a potential mechanism underlying the effect of reward on stress resilience. However, its role in the natural reward process and its impact on stress resilience need more exploration.

The current study aimed to observe the effect of different intensity levels of stress on depression-like behavior in mice and to further explore the possible effect of reward on depression-like behavior during chronic social defeat stress and potential cerebral mechanisms (especially ECs and mGluR5) underlying this effect. Our hypotheses were as follows: (1) stress may induce more depression-like behavior under high-stress intensity; (2) reward might enhance stress resilience and reduce depression-like behavior, with a more significant effect under high-stress intensity; (3) and reward may render the neural damage of stress through regulating the expression level of ECs and the mGluR5 in VTA and DRN under high-stress intensity.

## 2. Materials and methods

### 2.1. Animals

Animal care and use conformed to the institutional guidelines of the National Institute of Biological Sciences, Beijing, as well as the governmental regulations of China. Adult male C57BL/6J mice (9–12 weeks) and CD-1 mice (4–6 months) were used. Mice were housed individually for 2–3 weeks before modeling at a temperature of 22–25°C on a reverse 12-h light/dark cycle (lights on at 9:00 p.m.) with a standard chow diet. Drinking water was freely accessible unless noted otherwise. Mice were grouped into a large-stress group, a small-stress group, and a no-stress control group.

### 2.2. Chronic social defeat stress (CSDS)

**Figure 1:**
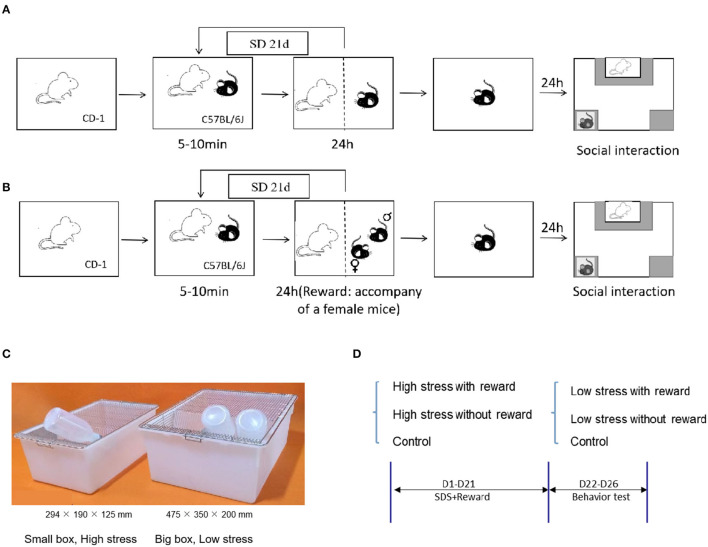
Experiment protocol. **(A)** Schematic representation of the CSDS. **(B)** Schematic representation of the reward during CSDS: 24-h accompany of female mice. **(C)** Schematic representation of the different sizes in the cage for large and small stress. **(D)** Experimental flowchart, including grouping and processing, and modeling information.

Based on the standard modeling procedure of literature (52), C57 mice were placed into the cage of CD-1 attacking mice for 10 min every day for sustained 21 days during continual modeling (53, 54)^1^. Then the C57 was put into the other side of the cage with a transparent partition for the rest of the day, in which C57 could smell and see CD-1 but without contact. C57 mice were exposed to a newly attacked CD-1 every day to avoid C57 mice adapting to CD−1. The weights of C57 mice were measured every day. Refer to Figure 1A for details about the experiment protocol.

### 2.3. Reward

During 21-day chronic social defeat stress, 24-h accompany of a female mouse was given as a social and sexual reward. That is, except 10 min of being attacked, male C57 mice were placed on the other side of the transparent partition and companied by a prolific and healthy female C57 mouse for the rest of the day (26) (Figure 1B).

### 2.4. Intensities of stress

**Figure 2:**
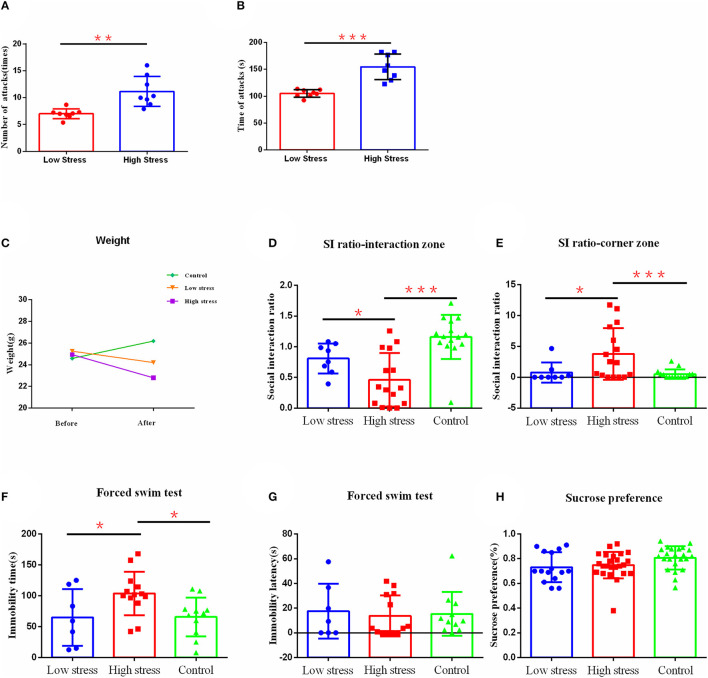
The effect of different intensities of stress on depression-like behavior. **(A)** The number of mice attacked per day for large and small stress. **(B)** The number of times mice were attacked per day for large and small stress. **(C)** Body weight. **(D)** Social interaction ratio in the interaction zone. **(E)** Social interaction ratio in corner zone. **(F)** Immobility time. **(G)** Immobility latency in the forced swim test. **(H)** Sucrose preference. ^*^p < 0.05, ^**^p < 0.01, and ^***^p < 0.001. Low, under the low stress (big cage), *n* = 8; high, under the high stress (small cage), *n* = 15; control, *n* = 15. Error bars in figures represent S.D.

To differ the severity levels of stress, we utilized different sizes of mice cages during CSDS. Modeling in the small cage (294 × 190 × 125 mm) represents a large amount of stress, in which mice have less escape space while modeling in the large cage (475 × 350 × 200 mm) represents a small amount of stress (Figure 1C), in which mice have more space to escape and are less likely to be attacked. See Figure 1D for details about the experimental flowchart. Refer to Figures 2A, B for details about the time and frequency of attack of large and small stress. The control group did not receive any attack during 21-day modeling (52).

### 2.5. CB1 agonists and antagonist

To observe the effect of different levels of CB1 on the depression-like behavior of mice, after everyday social defeat stress, CB1 agonists (URB-597, 1 mg/kg, MCE, USA) or inhibitors (AM251, 3 mg/kg, MCE, USA) was intraperitoneally injected into mice; only the control group received an injection of the same amount of saline.

### 2.6. Behavior test

After modeling, all animals were tested with behavior tests.

#### 2.6.1. Social interaction test

The test was divided into two stages, and each stage lasted for 2.5 min. In the first stage, the empty cage was placed into the social interaction zone, and in the second phase, an aggressive CD-1 was put into the cage at the social interaction zone. The C57 mice were put into the test zone, and the time and trajectory of C57 mice staying at the social interaction and corner zone were recorded. The social interaction index (SI) of the social interaction and corner zone was calculated, i.e., the time duration ratio of the second phase to the first phase at the interaction zone or the corner zone (52) (Figure 1A), which was also referred in the calculation of the ratio of susceptible/resilient animals following the social defeat stress. Resilience was defined by a ratio >1 of the time spent in the interaction zone during phase 2 over phase 1 with an interaction time longer than 60 s with the CD1 during phase 2. Conversely, susceptibility was defined as a ratio under 1 and an interaction time of <40 s with the CD1 during phase 2.

#### 2.6.2. Sucrose preference test

Sucrose Preference Test was used to assess the level of anhedonia in animals. After modeling, C57 mice were given two bottles of water to drink for 24 h. After that, one tap water bottle was replaced with a bottle containing 1% sucrose, changing the position of the two water bottles once a day for 3 days, and the proportion of sucrose consumption in total liquid consumption represented an indicator of sucrose preference (55).

#### 2.6.3. Force swimming test

The forced swimming test was used to assess despair behavior. In this test, C57 mice were placed in a transparent cylindrical barrel (50 cm in height and 11 cm in diameter) with 17 cm deep water (water temperature 25 ± 1°C), which was changed before testing the next mouse. Animals' behavior in water was recorded by camera for 6 min, and the immobile time and the latency to the first immobility were calculated in the last 4 min. Immobility was defined as floating and small strokes to keep the head out of the water only (55).

### 2.7. qPCR

After modeling and behavioral tests, only animals under high stress were further analyzed with cerebral biomarkers. Mice were sacrificed after behavioral experiments through intraperitoneal injection of pentobarbital sodium. The brain was removed quickly after decapitation and placed on an ice glass dish. Total RNA was extracted from frozen VTA and DRN samples for D1, D2, CB1, and mGluR5 analyses. RNA concentrations were ascertained by absorbance at 260 nm, and the purity was evaluated from the A260/A280 ratio value using the Nanodrop spectrophotometer (Wilmington, DE). According to the manufacturer's instructions, RNA (1,000 ng) from each sample was reverse transcribed to cDNA using RevertAid™ First-Strand cDNA Synthesis Kit (#K1621, Formentas, USA). RT-PCR was performed in a Bio-Rad IQ™ system (Bio-Rad, Hercules, USA) using SYBR Green Q-PCR analysis. Primer sequences were DRD1-F: TGGTCTGTGCTGCCGTTATCAG; DRD1-R: CAATCTCAGCCACTGCCTTCCA; DRD2-F: CCTGTCCTTCACCATCTCTTGC; DRD2-R: TAGACCAGCAGGGTGACGATGA; mGluR5-F: ACCAACCAACTGTGGACAAAG; mGluR5-R: CAAGAGTGTGGGATCTGAATTGA; CB1-F: TCTTGTGAGAATTGGTTGGCAA; CB1-R: CATCTCCATAGGTCCCCTTATCA; GAPDH-F: AATGGATTTGGACGCATTGGT; and GAPDH-R: TTTGCACTGGTACGTGTTGAT. The CB1 and mGluR5 gene levels were normalized for each well of the GAPDH mRNA calculated by the ΔΔCT method. Each sample was analyzed in triplicate.

### 2.8. Western blot

Western blotting was used to test the protein levels of CB1, mGluR5, and Glyceraldehyde-3-phosphate dehydrogenase (GAPDH) in the VTA and DRN of mice. DRN and VTA tissue punches were lysed in RIPA buffer (Beyotime Biotechnology, China) containing protein inhibitors (1 mM Phenylmethanesulfonyl fluoride (PMSF), Roche, USA). An amount of 30 mg supernatant protein for each sample was electrophoresed on precast 10% SDS gradient gels, followed by electro-blotting onto polyvinylidene fluoride (PVDF) membranes. PVDF membranes were washed in tris-buffered saline with 0.1% Tween-20 (TBS-T) and blocked in 5% BSA for 2 h at 25°C, and then incubated in antibodies for CB1 (1:1,000, Proteintech, China), mGluR5 (1:1,000, Millipore, USA), and GAPDH (1:10,000, Proteintech, China) overnight at 4°C, peroxidase-labeled secondary antibody at 25°C for 1 h, and bands were visualized by enhanced chemiluminescence. Serum for enzyme immunoassays (EIAs) was obtained from centrifuged trunk blood, and steroid levels were assayed with commercially available EIA kits according to the manufacturer's instructions. The gray value of immune reactivity was imaged and calculated using the ImageJ software.

### 2.9. Liquid chromatography coupled to tandem mass spectrometry (LC-MS/MS)

#### 2.9.1. Lipid extraction from tissue

For the analysis of endocannabinoid content, brain regions were subjected to a lipid extraction process. Tissue samples were weighed (usually 10–25 mg) and placed into EP tubes, 1 ml of ice-cold saline containing 100 μm PMSF, with 50 μl of [d8] anandamide (AEA) (40 ng/ml) and 50 μl of [d8]2-arachidonoylglycerol (2-AG) (1,000 ng/ml) were added for extraction. Tissue was homogenized with a homogenizer (Eppendorf Thermomixer, German). Then the tissue homogenate was mixed with ethyl acetate/hexane (9:1, v/v) and vortexed for 5 min. Samples were incubated overnight at −20°C to precipitate proteins and then centrifuged to remove particulates (14,000 × *g* for 5 min at 4°C). The supernatants were removed to a new EP tube, and the process was repeated once more to obtain a particulate-free supernatant. This supernatant was then evaporated to dryness under N2 gas. Finally, lipid extracts were suspended in 50 μl of acetonitrile and stored at −80°C until analysis.

#### 2.9.2. Mass spectrometric detection of endocannabinoids

Liquid chromatography-mass spectrometry (LC-MS/MS) analyses were carried out on an Agilent Technologies 1200 series coupled with an Agilent Technologies 6410 Triple Quad LC/MS, installed with an Agilent Mass Hunter Workstation (Agilent Technologies, USA). The LC was equipped with a temperature-controlled CTC autosampler. An Agilent Eclipse Plus C18 column (2.1 × 150 mm, 3.5 μm particle diameter) was used. Samples were analyzed isocratically, at a flow rate of 250 μl/min and a solvent composition of 25% mobile phase A (0.2% formic acid), and 75% mobile phase B (acetonitrile). The LC column was maintained at 30°C and the samples at 10°C.

The mass spectrometer was operated in Electrospray Interface (ESI), MRM Mode, with the ion-spray voltage set at 5,500 V, and source temperature at 340°C. Protonated molecular ions of AEA (m/z 348) and AEA-d8 (m/z 356) and ammonium adduct ions of 2-AG (m/z 396) and 2-AG-d8 (m/z 404) were selected as the respective precursor ions for CID. MRM scan modes were used with Q1 and Q3 both at unit resolution. Optimized collision energies for the transitions were as follows: AEA (348 to 62) CE 22V; AEA-d8 (356 to 62) CE 22V; 2-AG (396 to 287) CE 15V; and 2-AG-d8 (404 to 294) CE 17V.

AEA and 2-AG standard stock solutions were diluted with acetonitrile to obtain standard solutions of different mass concentrations. The concentrations were as follows: AEA concentrations of 125, 62.5, 12.5, 2.5, 0.50, and 0.1 ng/ml, 2-AG concentrations of 1,250, 625, 125, 25, 5, and 1 ng/ml, and 200 μl of physiological saline in which 10 mmol PMSF was dissolved. A volume of 25 μl each of AEA-d8 and 2-AG-d8 internal standard solution was added, followed by 800 μl of ethyl acetate, mixed with n-hexane (9:1, v/v), vortexed, centrifuged, and then air-dried with nitrogen. A volume of 100 μl of acetonitrile was added to reconstitute, and 5 μl of which was then injected to make a standard curve.

### 2.10. Statistics

Data were presented as mean ± S.D. One-way analysis of variance (ANOVA) with Tukey's *post hoc* test was carried out to compare the behavioral and cerebral variables of three groups (high-stress, low-stress, and control group). An independent *t*-test was conducted to compare the behavioral and cerebral differences between the two groups (with and without reward). Differences were considered as significant if *p* < 0.05. All analyses were performed using SPSS19.

## 3. Results

### 3.1. Stronger stress led to higher degrees of depression-like behavior in mice

To test whether different intensities of stress lead to different degrees of depression-like behavior in mice, we applied different intensities of stress on mice by utilizing different sizes of mice cages during CSDS, in which a big cage (475 × 350 × 200 mm) represented a small amount of stress (more escape space and a lower possibility of being attacked), and a small cage (294 × 190 × 125 mm) represented a large amount of stress (less escape space and a higher possibility of being attacked). Refer to Figure 1C for details. To verify that this method could distinguish between different intensities of stress, we recorded the numbers and times that mice were attacked per day during modeling. We found that in the small cage, mice did receive more attacks, referring to attack numbers (Figure 2A) or times (Figure 2B), compared with the big cage. Meanwhile, during the modeling process, we also objectively recorded changes in body weight and found that different stress intensities also had significant effects on body weight. There were no differences in weight between groups before modeling. However, after modeling, weights of the high stress group (*t* = 3.909, *p* < 0.001) reduced significantly, while there was no significant difference in the low stress group, and the control group significantly increased in weight after modeling (*t* =−5.621, *p* < 0.001) (Figure 2C).

After 21 days of social defeat stress modeling, the depression-like behavior was observed through social interaction test, sucrose preference test, and force swimming test. Results found that the ratio of susceptible/resilient animals following social defeat stress under low stress was 6:2 (Supplementary Figure 1A), while under high stress was 13:2 (Supplementary Figure 1B). There were significant differences in the social interaction index between groups in the social interaction area [*F*_(2, 35)_ = 13.143, η^2^ = 0.75, *p* < 0.001]. Specifically, the social interaction index of mice in the social interaction area under high stress was significantly smaller than that in the low stress group (*p* = 0.040) and the control group (*p* < 0.001) (Figure 2D). Similarly, there were significant differences in the social interaction index between groups in the corner area [*F*_(2, 36)_ = 5.996, η^2^ = 0.25, *p* = 0.006], in which the high stress group had significantly higher index scores compared with the low stress group (*p* = 0.019) and the control group (*p* = 0.003) (Figure 2E). The high stress group also showed longer immobility time in the forced swimming test [*F*_(2, 28)_ = 4.105, η^2^ = 0.23, *p* = 0.027], compared with the low stress group (*p* = 0.033) and the control group (*p* = 0.017) (Figure 2F). However, there was no difference between groups in the immobility latency [*F*_(2, 28)_ = 0.100, η^2^= 0.01, *p* = 0.906] (Figure 2G) and sucrose preference [*F*_(2, 59)_ = 1.493, η^2^= 0.09, *p* = 0.242] (Figure 2H). No significant differences were confirmed between low stress and control groups (*p* > 0.05).

### 3.2. Reward reduced depression-like behavior and enhanced stress resilience in mice with CSDS with a stronger effect under high stress

**Figure 3:**
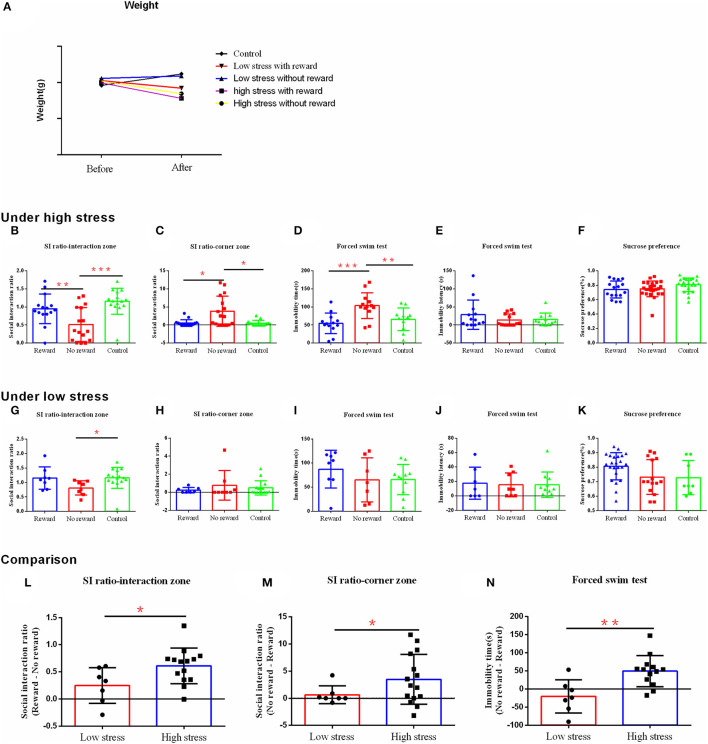
The effect of reward on depression-like behavior and stress resilience under the different intensities of stress. **(A)** Body weight. **(B–F)** The results of the behavior test under high stress. **(G–K)** The results of the behavior test under low stress. **(L–N)** Comparison of the difference between the reward group and the non-reward group under different stress intensities. **(B, G, L)** Social interaction ratio in the interaction zone. **(C, H, M)** Social interaction ratio in the corner zone. **(D, I, N)** Immobility time. **(E, J)** Immobility latency in the forced swim test. **(F, K)** Sucrose preference. ^*^p < 0.05, ^**^p < 0.01, and ^***^p < 0.001. Under high stress, reward group, *n* = 15; non-reward group, *n* = 16; control group, *n* = 15; under low stress, reward group, *n* = 7; non-reward group, *n* = 8; control group, n =15. Error bars in figures represent S.D.

To test the effect of reward on depression-like behavior under CSDS stress, we utilized reward (accompanied by a female mouse) on mice during the period of CSDS and compared the depression-like behavior after stress. Under both high and low stress groups, reward increased weight (Figure 3A).

Under high stress, the ratio of susceptible/resilient animals following social defeat stress with reward was 10:5 (Supplementary Figure 1C), while without reward was 13:3 (Supplementary Figure 1D). Reward changed the social avoidance behavior of mice [*F*_(2, 43)_ = 9.681, η^2^= 0.31, *p* < 0.001], in which the non-reward group showed significantly less social interaction behavior compared with the reward group (*p* = 0.006) and the control group (*p* < 0.001) (Figure 3B). Similarly, there were significant differences in the social interaction index between groups in the corner area [*F*_(2, 43)_ = 8.417, η^2^= 0.28, *p* = 0.001], with more social avoidance in the non-reward group compared with the reward group (*p* = 0.024) and the control group (*p* = 0.021) (Figure 3C). Moreover, the three groups differed significantly in the immobility time in the forced swimming test [*F*_(2, 34)_ = 8.446, η^2^= 0.33, *p* = 0.001], with longer immobility time in the non-reward group compared with the reward group (*p* < 0.001) and the control group (*p* = 0.006) (Figure 3D). However, the three groups did not differ significantly on the immobility latency [*F*_(2, 34)_ = 1.072, η^2^= 0.06, *p* = 0.354] (Figure 3E) and sucrose preference [*F*_(2, 60)_ = 1.472, η^2^= 0.08, *p* = 0.254] (Figure 3F). No significant differences were confirmed between the reward and control groups (*p* > 0.05).

Under low stress, the ratio of susceptible/resilient animals following social defeat stress with reward was 2:6 (Supplementary Figure 1E), while without reward was 6:2 (Supplementary Figure 1F). Reward only changed the social interaction behavior in mice [F _(2, 27)_ = 3.074, η^2^= 0.20, *p* = 0.062], with more social interaction behavior in the reward group (*p* = 0.053) and the control group (*p* = 0.027) compared with the non-reward group (Figure 3G). However, there was no significant difference between groups on the social interaction index in the corner area [*F*_(2, 37)_ = 0.642, η^2^ = 0.04, *p* = 0.534] (Figure 3H), the immobility time [*F*_(2, 23)_ = 0.890, η^2^ = 0.07, *p* = 0.424] (Figure 3I), latency [*F*_(2, 23)_ = 0.260, η^2^ = 0.01, *p* = 0.773] (Figure 3J) in a forced swimming test, and the sucrose preference [*F*_(2, 36)_ = 1.292, η^2^ = 0.13, *p* = 0.294) (Figure 3K).

To better compare the effect of reward on depression-like behavior after different intensities of stress, we calculated the score differences between reward and non-reward groups, which were included in further analysis. Analysis showed that under the high stress, the difference scores were significantly bigger on the social interaction index in the social interaction zone (*t* = −2.360, *p* = 0.017) (Figure 3L), and smaller on the social interaction index in the corner zone (*t* = −2.228, *p* = 0.038) (Figure 3M), and the immobility time of forced swimming (*t* = −3.330, *p* = 0.003) (Figure 3N), compared with those under the low stress. That is, under high stress, the reward had a greater effect to reduce the depression-like behavior than that of low stress.

### 3.3. The effect of reward and stress on the dopamine system in VTA and DRN

To confirm the effect of the reward on depression-like behavior, we further explored the expression of dopamine receptors [dopamine receptor D1 (DRD1) and dopamine receptor D2 (DRD2)], which was an important reward molecular mechanism, under high stress as previously described.

**Figure 4:**
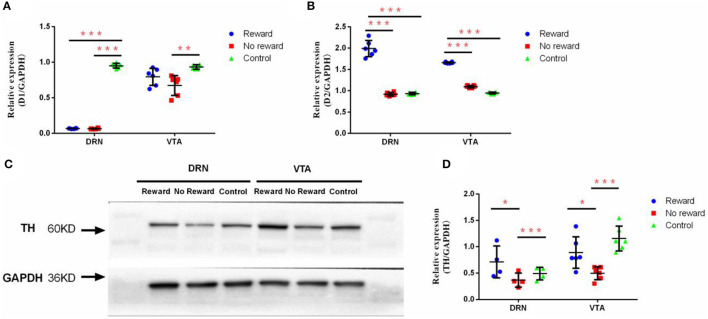
The effect of reward and stress on the dopamine system in DRN and VTA. **(A)** The mRNA expression of D1 in DRN and VTA, *n* = 9. **(B)** The mRNA expression of D2 in DRN and VTA, *n* = 9. **(C)** Western Blot analysis of the expression of TH in DRN and VTA. **(D)** Quantification of the expression level of TH in DRN and VTA, *n* = 6. ^*^p < 0.05, ^**^p < 0.01, and ^***^p < 0.001. Error bars in figures represent S.D.

The effect of reward on the mRNA expression of D1 and D2 in the DRN and VTA regions was observed by qPCR. Results found that in DRN, the expression level of D1 was significantly lower in the reward group and non-reward group compared with the control group (*p* < 0.001) (Figure 4A), while the D2 expression levels of the non-reward group and control group were significantly lower compared with the reward group (*p* < 0.001) (Figure 4B). Similarly, in VTA, the expression of D1 was significantly lower in the non-reward group compared with the control group (*p* < 0.001) (Figure 4A), while the reward group was not significantly different from the control group. The expression levels of D2 in the non-reward group and control group were significantly lower compared with the reward group (*p* < 0.001) (Figure 4B).

The effect of reward on the protein expression of TH in the DRN and VTA regions was examined through Western Blot (Figure 4C). Results showed that in DRN, the expression level of TH in the non-reward group was significantly lower compared with the reward group (*p* = 0.037) and the control group (*p* = 0.008) (Figure 4D). Similarly, in VTA, the expression level of TH in the non-reward group was significantly lower compared with the reward group (*p* = 0.010) and the control group (*p* < 0.001) (Figure 4D).

### 3.4. Potential ECs and mGluR5 mechanisms *via* neuroprotection in DRN and VTA underlying the effect of reward on stress resilience

To further explore whether neural ECs and mGluR5 underlie the effect of reward on stress resilience and depression-like behavior, we further examined the changes in the expression of ECs and mGluR5 in VTA and DRN and also observed the effects of CB1 agonists and antagonists on depression-like behavior under high stress conditions.

**Figure 5:**
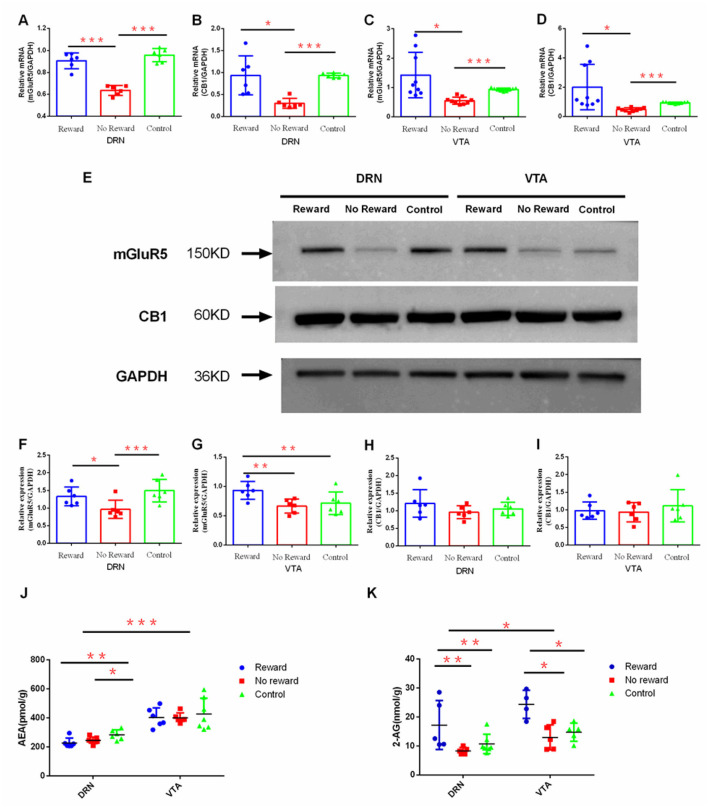
The effect of reward on the expression of ECs and mGluR5 in DRN and VTA. The mRNA expression of mGluR5 **(A)** in DRN, *n* = 6; **(B)** in VTA, n= 9; the mRNA expression of CB1; **(C)** in DRN n=6; **(D)** in VTA, *n* = 9; **(E)** Western blot analysis of the expression of mGluR5 and CB1; quantification of the expression level of mGluR5 **(F)** in DRN and **(G)** in VTA, *n* = 6; quantification of the expression level of CB1 **(H)** in DRN and **(I)** in VTA *n* = 6; **(J)** LC-MS/MS analysis of the expression of AEA in DRN (reward group, *n* = 6; non-reward group, *n* = 7; control group, n= 6) and VTA (reward group, *n* = 7; non-reward group, *n* = 6; control group, n= 6); **(K)** LC-MS/MS analysis of the expression of 2-AG in VTA (reward group, *n* = 4; non-reward group, *n* = 5; control group, n= 5) and DRN (reward group, *n* = 5; non-reward group, *n* = 5; control group, n= 7). ^*^p < 0.05, ^**^p < 0.01, and ^***^p < 0.001. Error bars in figures represent S.D.

The effect of reward on the mRNA expression of CB1 and mGluR5 in the DRN and VTA regions was observed by qPCR. Results found that in DRN, the expression level of mGluR5 was significantly lower in the non-reward group compared with the reward group (*p* < 0.001) and the control group (*p* < 0.001) (Figure 5A), while the CB1 expression level of the non-reward group was also significantly lower compared with the reward group (*p* < 0.001) and the control group (*p* < 0.001) (Figure 5B). Similarly, in VTA, the expression of mGluR5 was significantly lower in the non-reward group compared with the reward group (*p* = 0.018) and the control group (*p* < 0.001) (Figure 5C), while the expression level of CB1 in the non-reward group was significantly lower compared with the reward group (*p* = 0.046) and the control group (*p* < 0.001) (Figure 5D).

The effect of reward on the protein expression of CB1 and mGluR5 in the DRN and VTA regions was examined through Western Blot (Figure 5E). Results showed that in DRN, the expression level of mGluR5 in the non-reward group was significantly lower compared with the reward group (*p* = 0.041) and the control group (*p* = 0.006) (Figure 5F). In VTA, the expression level of mGluR5 in the reward group was significantly higher compared with the non-reward group (*p* = 0.011) and the control group (*p* = 0.033) (Figure 5G). However, the protein expression of CB1 in VTA and DRN was not significantly different between the three groups (Figures 5H, I).

The effect of reward on the expression of AEA and 2-AG in DRN and VTA regions was further examined by liquid chromatography coupled with tandem mass spectrometry (LC-MS/MS). Results showed that in DRN, the expression of AEA in the reward group (*p* = 0.006) and non-reward group (*p* = 0.034) was significantly lower compared with the control group (Figure 5J). The expression of AEA in VTA was not significantly different between the three groups. The expression of 2-AG in DRN was significantly higher in the reward group compared with the non-reward group (*p* = 0.014) and the control group (*p* = 0.043). Similarly, in VTA, the expression level of 2-AG in the reward group was significantly higher compared with the non-reward group (*p* = 0.002) and the control group (*p* = 0.005) (Figure 5K). Moreover, the expression of AEA (*t* = 8.299, *p* < 0.001) and 2-AG (*t* = 2.254, *p* = 0.032) in VTA was significantly higher compared with those in DRN in all three groups (Figures 5J, K).

**Figure 6:**
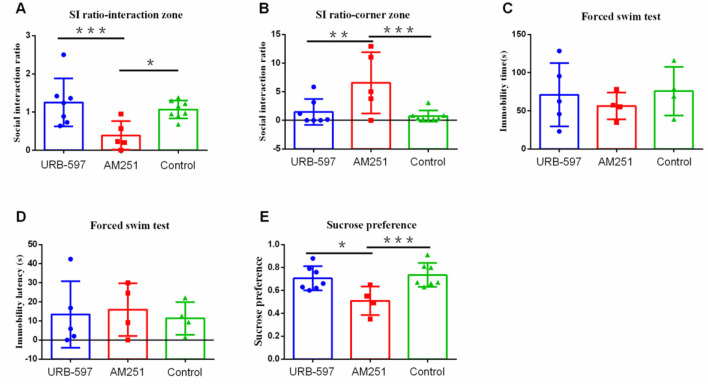
The effect of CB1 agonist and inhibitor receptors on depression-like behavior. **(A)** Social interaction ratio in the interaction zone (URB-597, *n* = 7; AM251, *n* = 5, Control, *n* = 8). **(B)** Social interaction ratio in the corner zone (URB-597, *n* = 7; AM251, *n* = 5, Control, *n* = 8). **(C)** Immobility time (URB-597, *n* = 5; AM251, *n* = 4, Control, *n* = 4). **(D)** Immobility latency (URB-597, *n* = 5; AM251, *n* = 4, Control, *n* = 4) in forced swim test. **(E)** Sucrose preference (URB-597, *n* = 7; AM251, *n* = 5, Control, *n* = 7). ^*^p < 0.05, ^**^p < 0.01, and ^***^p < 0.001. URB-597, CB1 agonist; AM-251, CB1 inhibitor. Error bars in figures represent S.D.

Furthermore, to confirm the role of CB1 in depression-like behavior, CB1 agonist (URB-597) and CB1 inhibitor (AM251) were injected intraperitoneally 10 min before the modeling under high stress intensities. After 21 days of modeling, the behavioral test found that the ratio of susceptible to resilient animals following social defeat stress under CB1 agonist (URB-597) was 3:4 (Supplementary Figure 1G), while under inhibitor (AM251), it was 5:0 (Supplementary Figure 1H). Compared with the CB1 agonist group (*p* = 0.004) and control group (*p* = 0.015), the CB1 inhibition group showed significantly less social interaction behavior in the social interaction zone (Figure 6A) as well as more social avoidant behavior in the corner area compared with the CB1 agonist group (*p* = 0.010) and control group (*p* = 0.003) (Figure 6B). However, the three groups did not differ significantly in the immobility time and immobility latency in forced swimming (*p* > 0.05) (Figures 6C, D). They further showed lower sucrose preference compared with the CB1 agonist group (*p* = 0.012) and the control group (*p* = 0.005) (Figure 6E).

## 4. Discussion

In this study, we observed the effect of different intensities of stress on depression-like behavior in mice and further confirmed the effect of reward on depression-like behavior during chronic social defeat stress and the potential neural mechanisms (especially ECs and mGluR5 in VTA and DRN) underlying this effect. The results helped reveal the effect of reward on chronic stressors and potential neural mechanisms, which might give implications for human beings.

This study tried to find a way to distinguish different intensities of stress through cage space in the social defeat stress for the first time. More attacks (in attack numbers or time) and decreased weight in the small cage indicated that our methods for dividing the stress intensity and reward method were effective, which needs more research to confirm. Our results indicated that stronger chronic social defeat stress led to higher degrees of depression-like behavior in mice. In human beings, based on interviews with 1,755 respondents, a study showed that a chronic response irritation (defined as sustained stress over 12 months) predicted more depressive symptoms compared with acute stress (4). Although the evidence is limited, the result combined with our result was powerful to suggest that in daily life, we should not only focus on intense acute stress but also pay attention to sustained chronic social defeat stress, especially with stronger intensity, which might cause severe continuous harm to people's mental health and play a role in the occurrence of emotional disorders such as depression.

In this study, we also confirmed that reward experiences (social and sexual rewards) could enhance stress resilience and reduce depression-like behavior in mice. Specifically, the reward group showed less depression-like behavior compared with the non-reward group, mainly manifested as more social interaction, less social avoidance, and less despair behavior. The results suggested that in the prevention and treatment of clinical depression, reward, specifically the combined social and sexual reward, might be considered a protective strategy. Previously, some studies already observed the effect of reward (i.e., sucrose, environmental enrichment, sweetened drink, food, and sexual activity of animals and self-affirmation and viewing appetitive pictures of people) on stress resilience (26–28, 56–59). However, in these studies, the reward was mainly non-social and given briefly or before stress, while the effect of combined social and sexual reward on depression-like behavior during chronic social defeat stress had not been observed, which was addressed in this study for the first time. In this study, female mice companionship was given as a social and sexual reward during 21 days of chronic social defeat stress and confirmed the effect of this reward on chronic social defeat stress during sustained stress. The reward used in this study contains both social and sexual reward, in which the social reward is moreand sexual reward is less (60). However, in clinic, human beings are more social with more social constraints (e.g., ethical). Thus, we should be very cautious to consider sex as a reward in clinical treatment of depression. This knowledge provides evidence to utilize combined social and sexual rewards in preventing severe outcomes under sustained chronic social defeat stress and offers new thinking for the prevention and treatment of depression in the clinic.

In addition, this study also confirmed that combined social and sexual rewards had a stronger effect in the reduction of depression-like behavior under high stress. In fact, another study has found that fluoxetine and desipramine reversed lower, but not higher, stress-induced brain reward deficits in susceptible rats (61), which was different from our results. The possible reason might be the differences in intervention strategies, i.e., medicine in the previous studies and combined social and sexual reward in this study. Moreover, in the chronic social defeat model used in this study, low stress caused fewer depression-like behaviors, which left less space for reward to show an effect, while high stress induced more severe depression-like behaviors, in which the protective effect of reward might be more obvious. This knowledge suggests that rewards should be used for more severe symptoms of depression in the clinic.

This study further explored potential brain mechanisms of reward underlying the effect of reward on stress resilience and found that the mRNA and protein expression of mGluR5 was decreased by chronic social defeat stress in VTA and DRN, which was reversed by reward. Previous studies referring to the connection between mGluR5 and reward mainly come from drug abuse studies, which found that mGluR5 mediated the behavioral response to cocaine and also participated in the expression and recovery of the reward regulation of alcohol (51), while the role of mGluR5 in the natural reward process was not explored. With the combined social and sexual reward, this study suggested that mGluR5 expression was increased by reward, especially in VTA, which indicated that mGluR5 might play a crucial role in combined social and sexual reward. Moreover, the mGluR5 was also closely related to stress, but there were some controversies and uncertainties about the role of mGluR5 in stress and reward in previous studies. One study found that mGluR5 (–/–) [also known as Grm5(–/–)] mice displayed more depression-like behaviors (e.g., learned helplessness, social withdrawal, and anhedonia) than control mice following exposure to various stressful stimuli, while several other studies found that mGlu5 receptor antagonism was associated with antidepressant-like effects and that mGlu5 receptor knockout mice displayed an antidepressant-like behavioral phenotype (50, 62). This study added some evidence to the former and further indicated the role of mGluR5 in the effect of reward on stress, i.e., stress reduces the expression level of mGluR5, which could be reversed by reward, indicating that mGluR5 may be a key mechanism underlying the effect of reward on stress resilience.

In terms of ECs, findings showed that the mRNA expression of the CB1 receptor was reduced by stress, which was enhanced by reward. Meantime, CB1 agonists reduced depressive-like behavior, while CB1 antagonists increased depressive-like behavior. This was consistent with our hypothesis because there was a close relationship between ECs and social reward (63–65). mGluR5 and CB1 can act together to promote neuroprotection (46); these two receptors work cooperatively to trigger the activation of cell signaling pathways to promote neuronal survival, which involves MEK/ERK1/2 and PI3K/AKT activation (45). Our results suggested that the neuroprotection of CB1 and mGluR5 plays a vital role in both stress and reward processes, which might be a crucial molecular mechanism underlying the effect of reward on stress resilience. This knowledge suggests that these receptors might be the potential targets for future medicines designed to treat stress-related diseases such as depression.

In addition, findings showed that reward and stress had different effects on the two ligands: AEA and 2-AG in the ECs. The content of AEA in DRN was significantly lower in the reward and non-reward group compared with the control group, while there was no significant difference between groups in VTA, indicating that stress significantly reduced the content of AEA in DRN, which could not be reversed by reward. The results were consistent with previous findings (66), which suggested that increasing the amount of AEA in the brain might be a way to prevent and treat depression. However, regarding the results of 2-AG in VTA and DRN, this is not entirely consistent with existing evidence, as most existing studies have shown that the increase in 2-AG content is a response to stress (67–69), while a few studies also showed that the expression of 2-AG in stress-like phenotypes decreased (70). In this study, stress did not significantly change the expression level of 2-AG in VTA and DRN. The possible reason might be that acute stress was commonly used in the above studies, which caused an increase in 2-AG expression levels, while chronic social defeat stress was used in this study, which resulted in different expression patterns of 2-AG. Furthermore, we also found that giving rewards during stress increased the content of 2-AG in VTA and DRN. This might be because our reward was accompanied by female mice, which might include rewards of sexual and social interactions, in which 2-AG was involved in the human sexual response cycle and played a role in the rewarding consequences of sexual arousal and orgasm (71). In summary, we believe that the increase in the content of 2-AG in VTA and DRN is involved in reward and that a possible mechanism underlying the effect of reward on stress resilience. These findings warrant more exploration on the expression level of 2-AG under acute and chronic social defeat stress in the future in order to make the result more decisive.

However, this study also had several limitations. First, the expression of CB1 receptors at the mRNA and protein levels was not consistent, which needs further exploration. Second, in this study, neither stress nor reward affected sucrose preference, indicating that social defeat stress and combined social and sexual reward mainly changed the social interaction behavior and despair behavior in mice but not the anhedonia behavior. This might be because both stress and reward were social, which had the most significant impact on social behavior. Moreover, the sucrose preference was influenced by CB1 agonists and antagonists, which confirmed our hypothesis in a sense.

## 5. Conclusion

This study observes the effect of different intensities of stress on depression-like behavior in mice under different intensities of CSDS and further confirms the effect of reward in reducing depression-like behavior during chronic social defeat stress and the potential cerebral mechanisms (especially neural ECs and mGluR5) underlying this effect. We clarify that rewards can be an effective way to improve stress resilience in daily life, which could be further practiced in human beings. The findings that stress reduces the expression of CB1 and mGluR5 in VTA and DRN and reward can reverse it suggest that the ECs and mGluR5 of VTA and DRN may be potential molecular mechanisms underlying the effect of reward on stress resilience, which might be potential targets of future medicines. These findings provide a new way of thinking about the prevention and treatment of clinical depression.

## Data availability statement

The original contributions presented in the study are included in the article/Supplementary material, further inquiries can be directed to the corresponding author.

## Ethics statement

The animal study was reviewed and approved by the Animal Ethics Committee of the Army Medical University.

## Author contributions

PS conducted most of the research, analyzed the data, and drafted the manuscript. LH conducted part of the research. QD designed the experiment. QD and HR helped in conducting the research and revised the manuscript. All authors contributed to the article and approved the submitted version.

## Footnotes

^1^Based on standard modeling procedure of literature (52), 7 - 10 days social defeat stress is a classic version of social defeat model. However, previous studies have suggested that 21 days defeat protocol is a chronic stress and more conducive to the occurrence of depression-like behavior (53, 54). This study aimed to explore possible effect of reward on depression-like behavior during chronic social defeat stress. We thus selected 21 days defeat protocol instead of 10 days in this study.

## Supplementary material

Supplementary Figure 1 The ratio of susceptible/resilient animals under different circulations. **(A, B)** The ratio of susceptible/resilient animals under low stress and high stress. **(C, D)** The ratio of susceptible/resilient animals with and without reward under high stress. **(E, F)** The ratio of susceptible/resilient animals with and without reward under low stress. **(G, H)** The ratio of susceptible/resilient animals under CB1 agonist (URB-597) and inhibitor (AM251).
