# Supplementary figures and images for: Reward enhances resilience to chronic social defeat stress in mice: Neural ECs and mGluR5 mechanism via neuroprotection in VTA and DRN
Source: Front Psychiatry. 2023 Feb 16;14:1084367. doi: 10.3389/fpsyt.2023.1084367 (PMC9978385; doi:10.3389/fpsyt.2023.1084367)

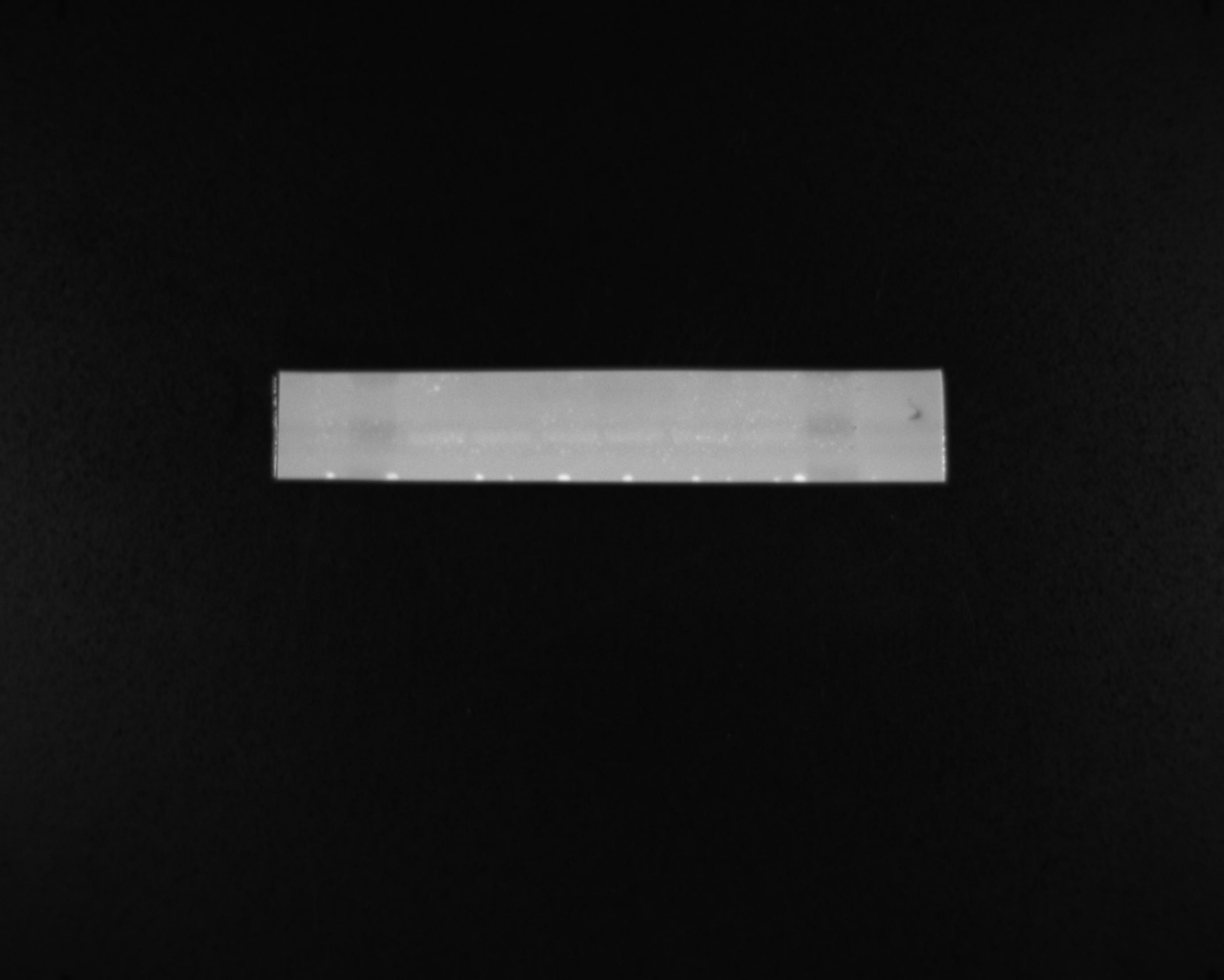

Supplement: Supplementary file 11 [file Data_Sheet_6.ZIP › CB1/CB1 1-1.tif]

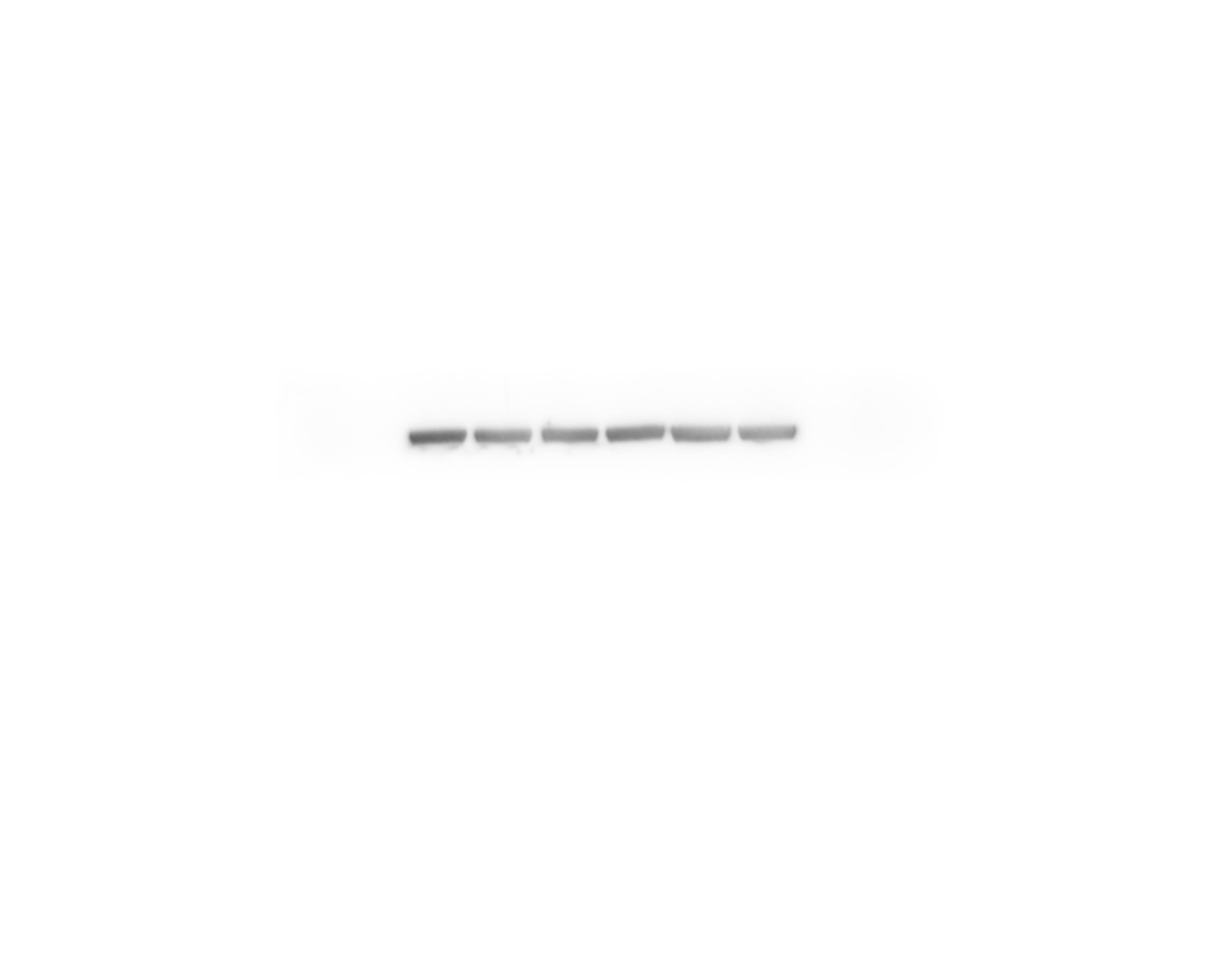

Supplement: Supplementary file 11 [file Data_Sheet_6.ZIP › CB1/CB1 1-2.tif]

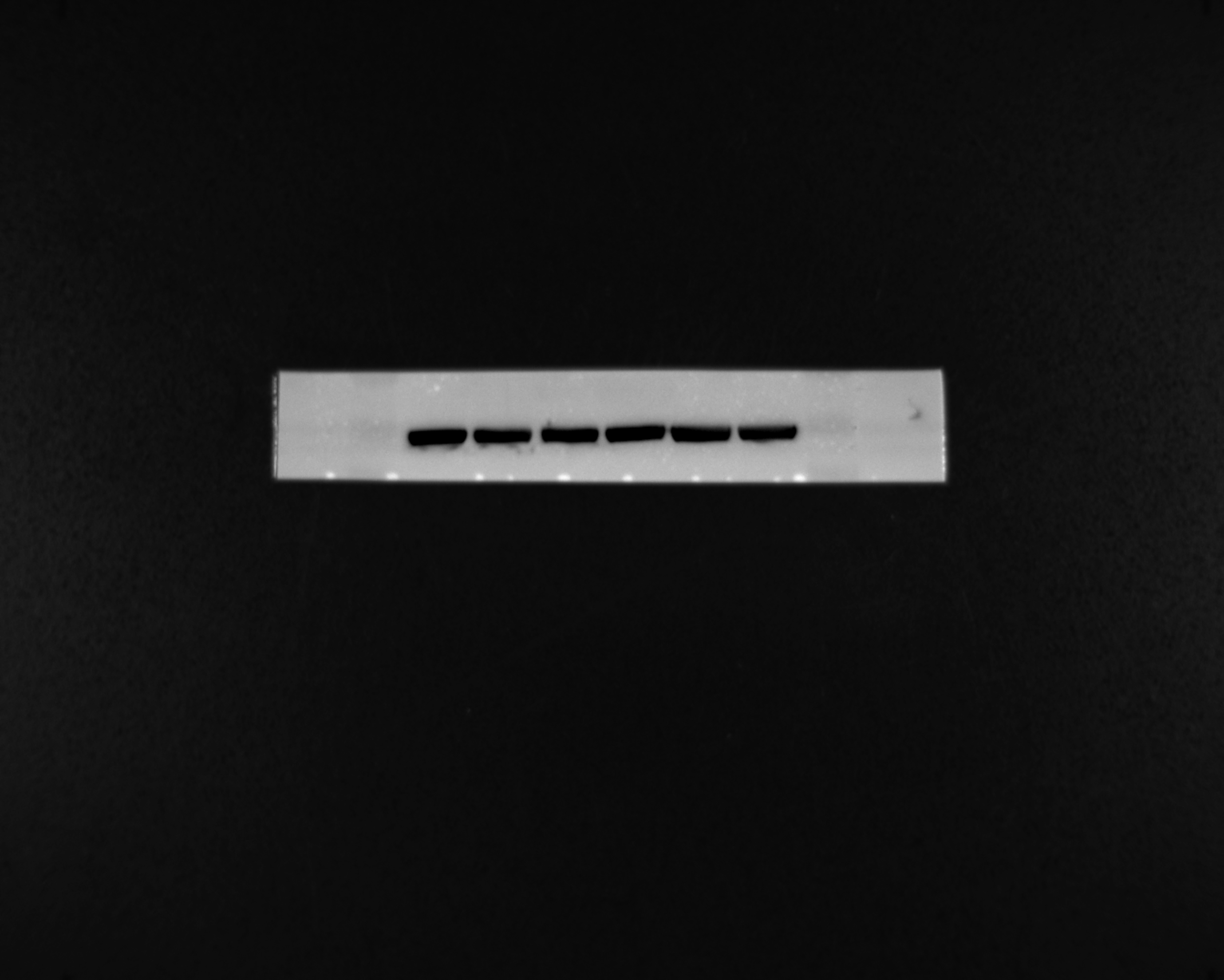

Supplement: Supplementary file 11 [file Data_Sheet_6.ZIP › CB1/CB1 1-3_.png]

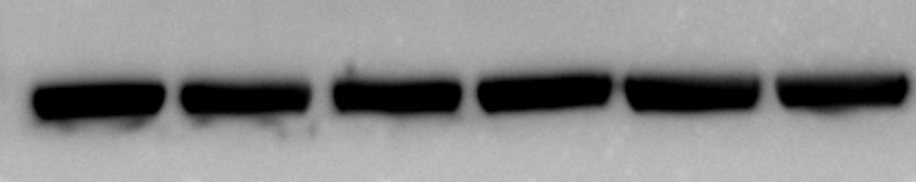

Supplement: Supplementary file 11 [file Data_Sheet_6.ZIP › CB1/CB1 1-3_wps图片_3.png]

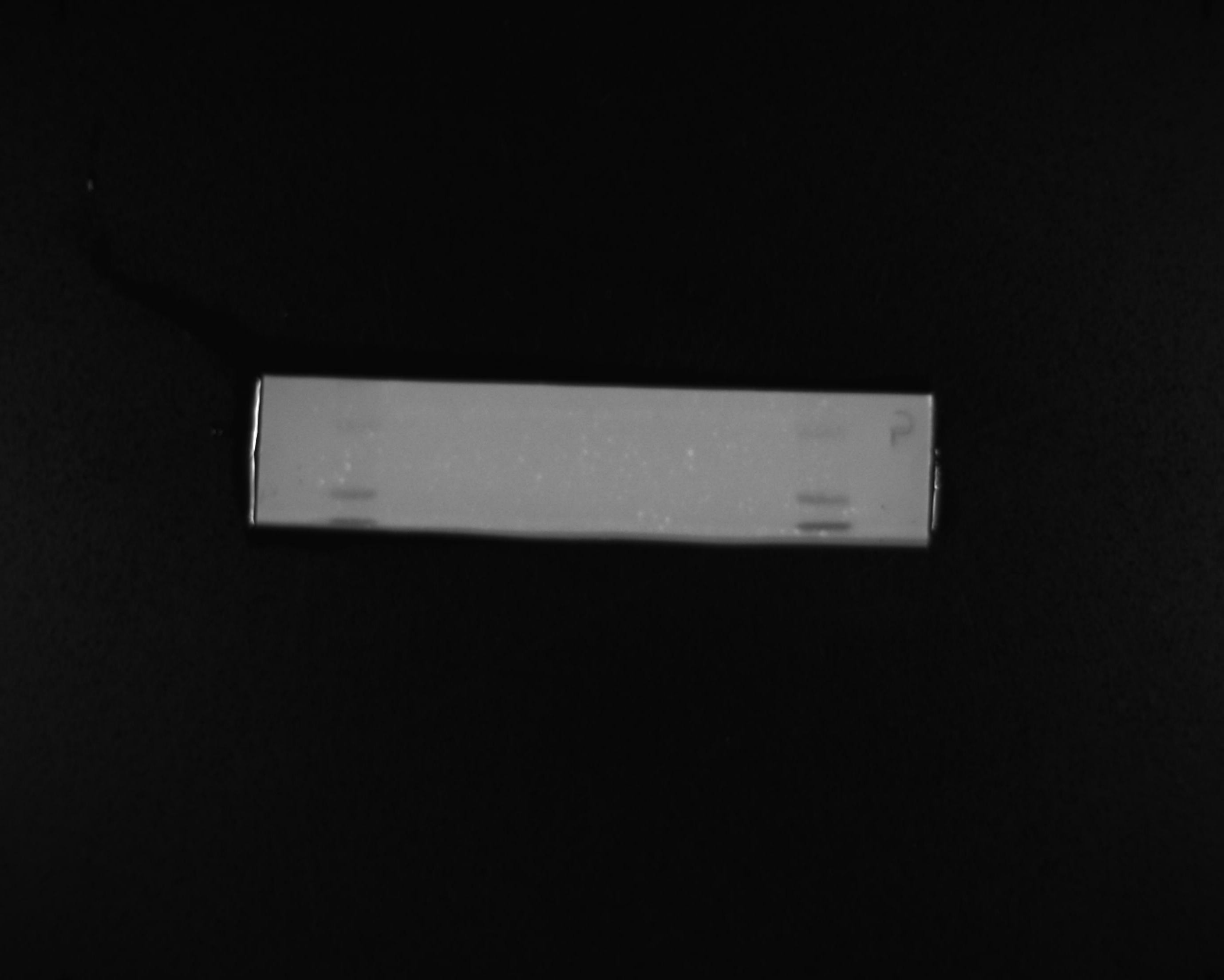

Supplement: Supplementary file 11 [file Data_Sheet_6.ZIP › GAPDH/GAPDH 1-1.tif]

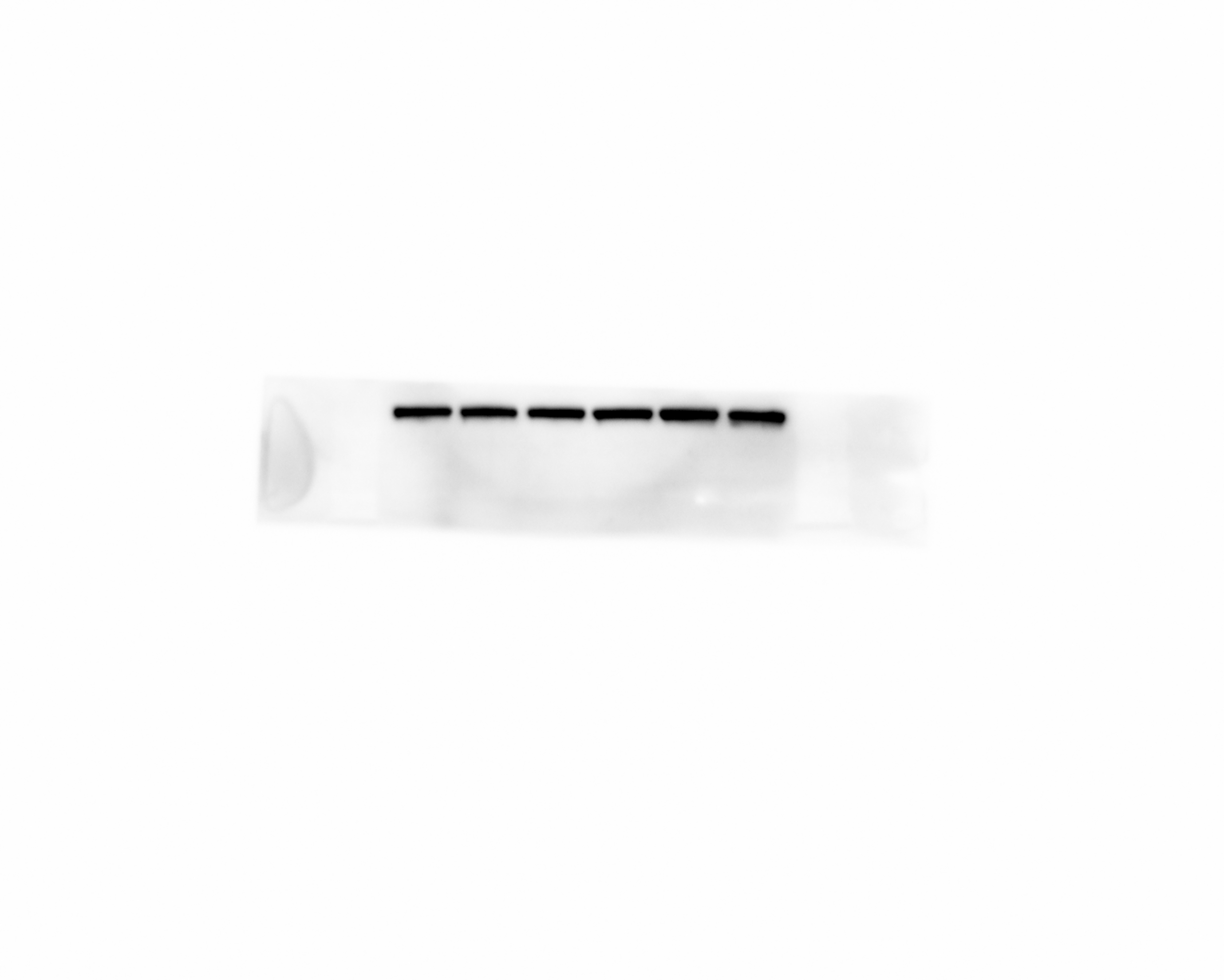

Supplement: Supplementary file 11 [file Data_Sheet_6.ZIP › GAPDH/GAPDH 1-2.png]

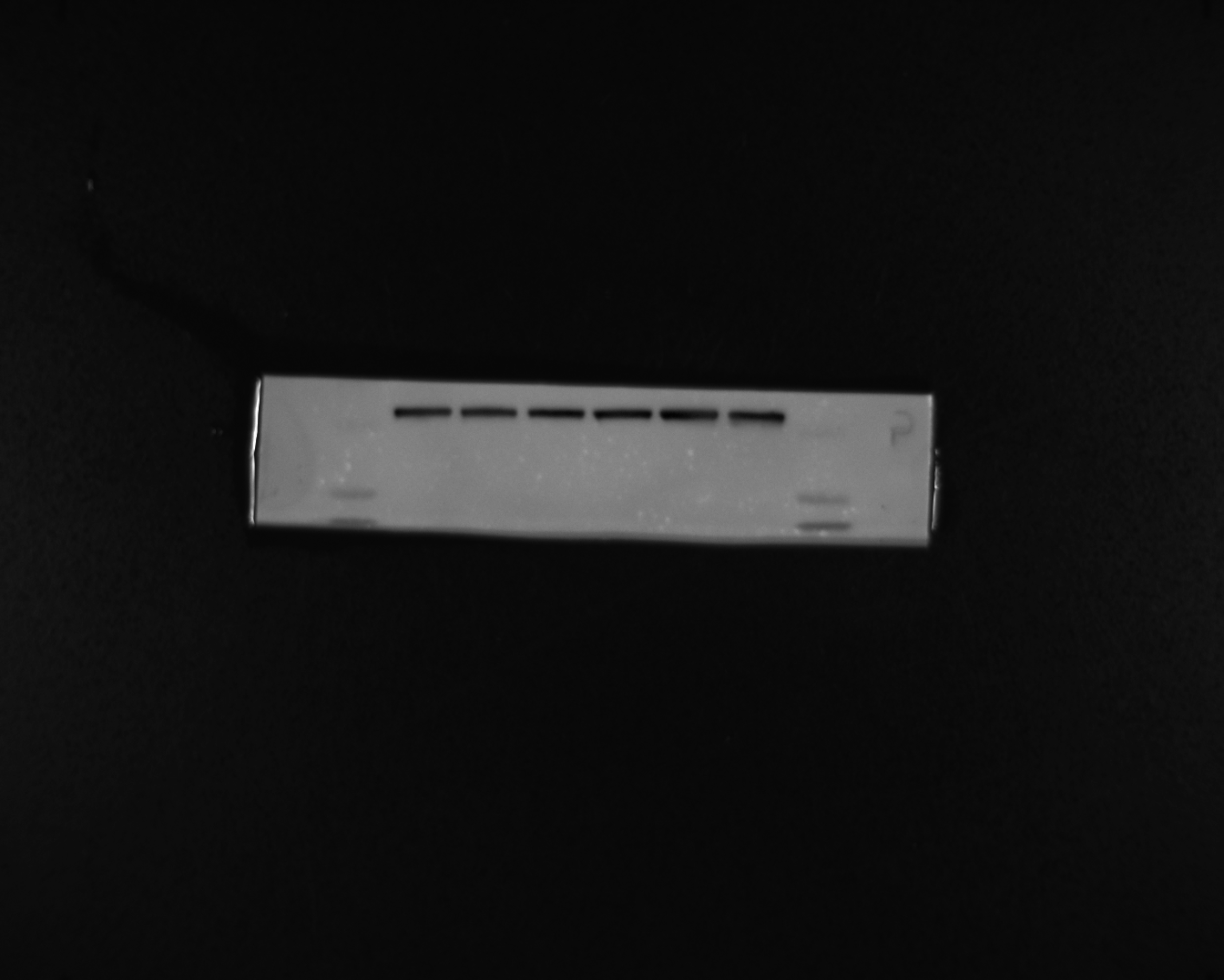

Supplement: Supplementary file 11 [file Data_Sheet_6.ZIP › GAPDH/GAPDH 1-3.png]

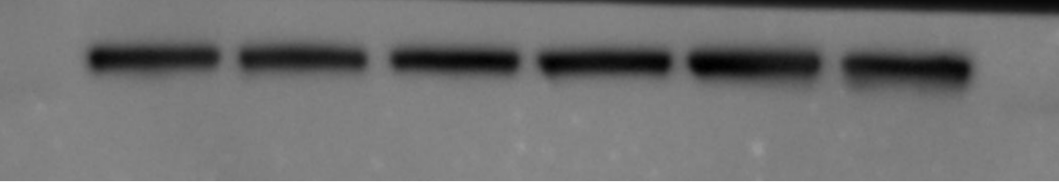

Supplement: Supplementary file 11 [file Data_Sheet_6.ZIP › GAPDH/WPS图片编辑.png]

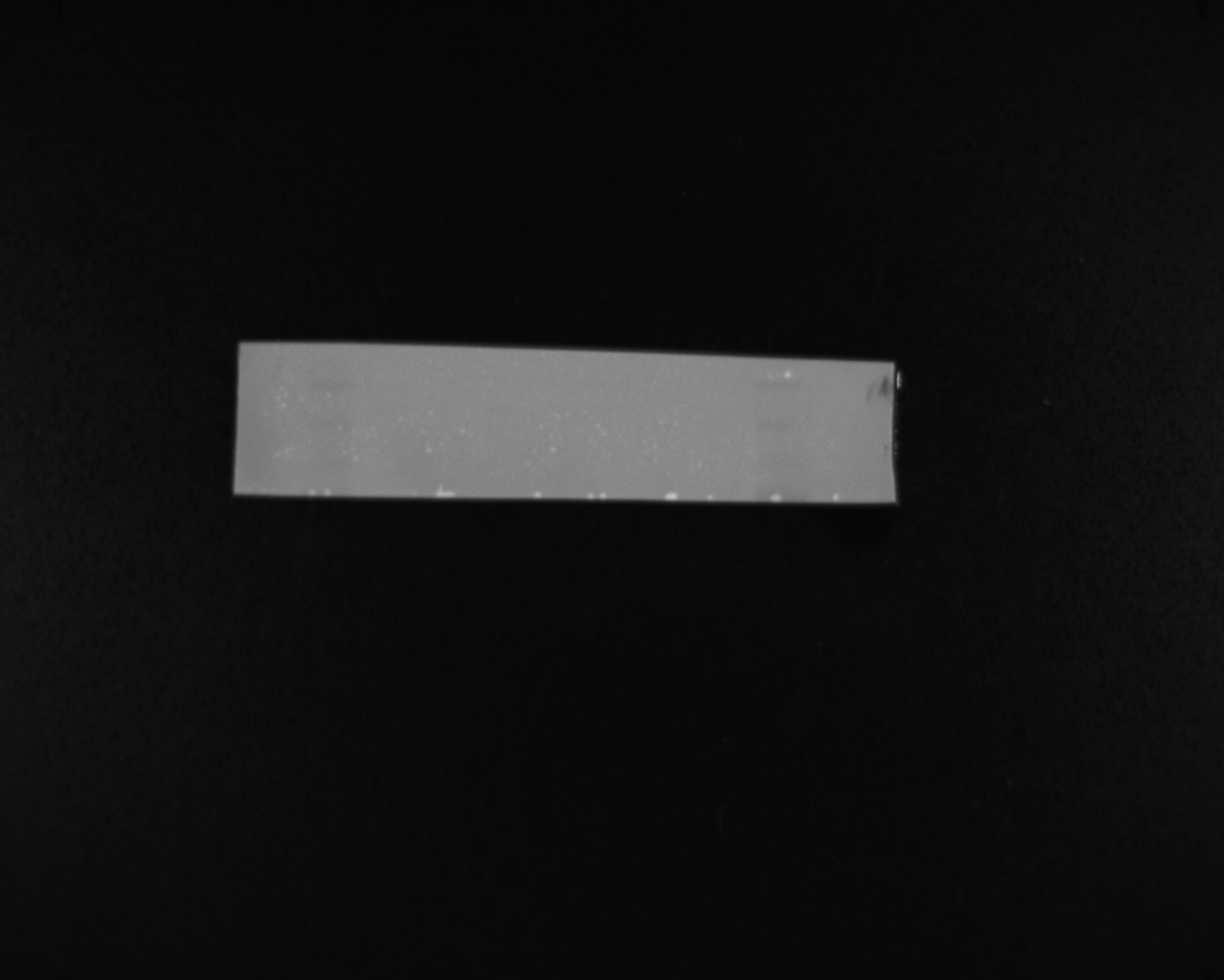

Supplement: Supplementary file 11 [file Data_Sheet_6.ZIP › mGluR5/mGluR5 1-1.tif]

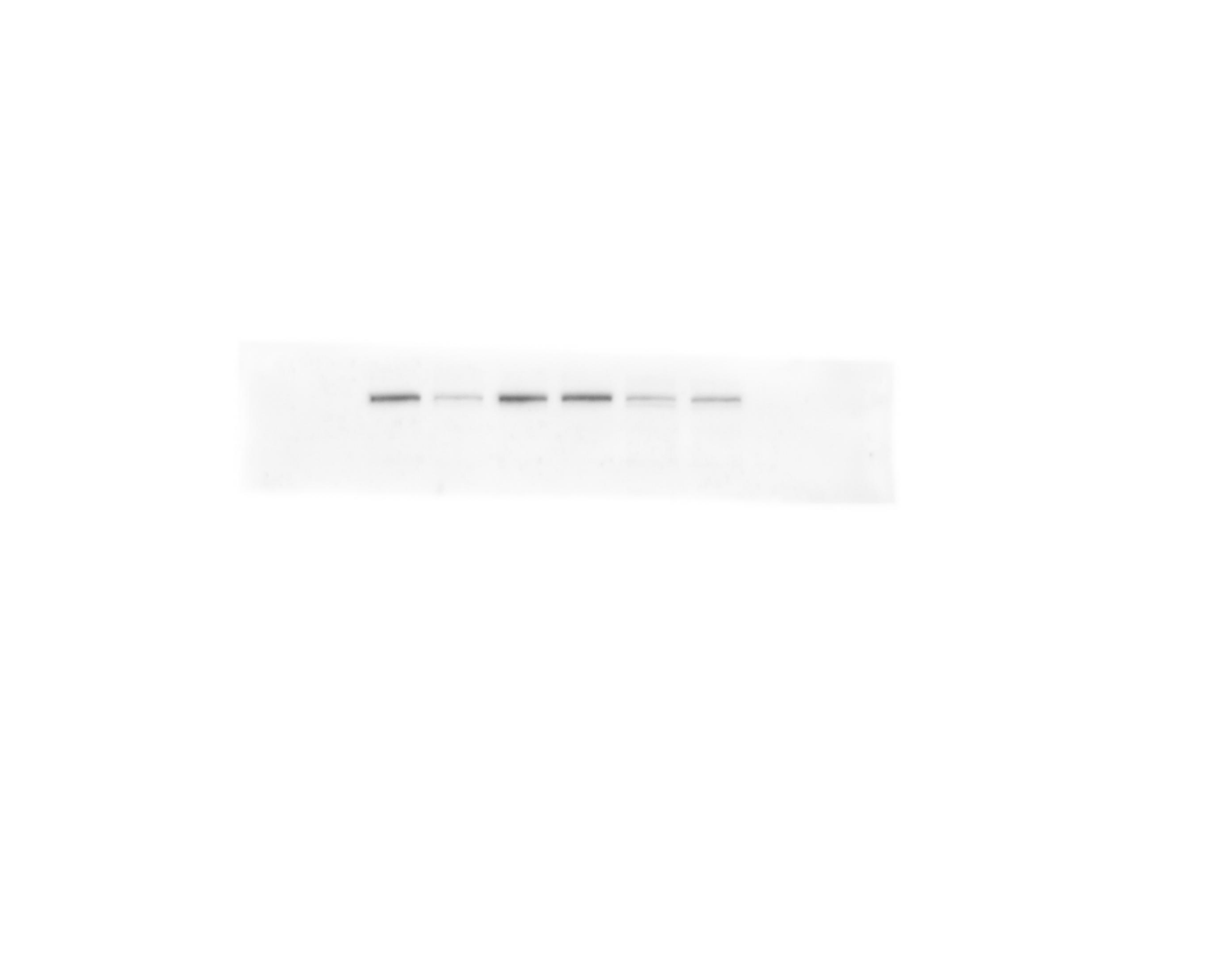

Supplement: Supplementary file 11 [file Data_Sheet_6.ZIP › mGluR5/mGluR5 1-2.tif]

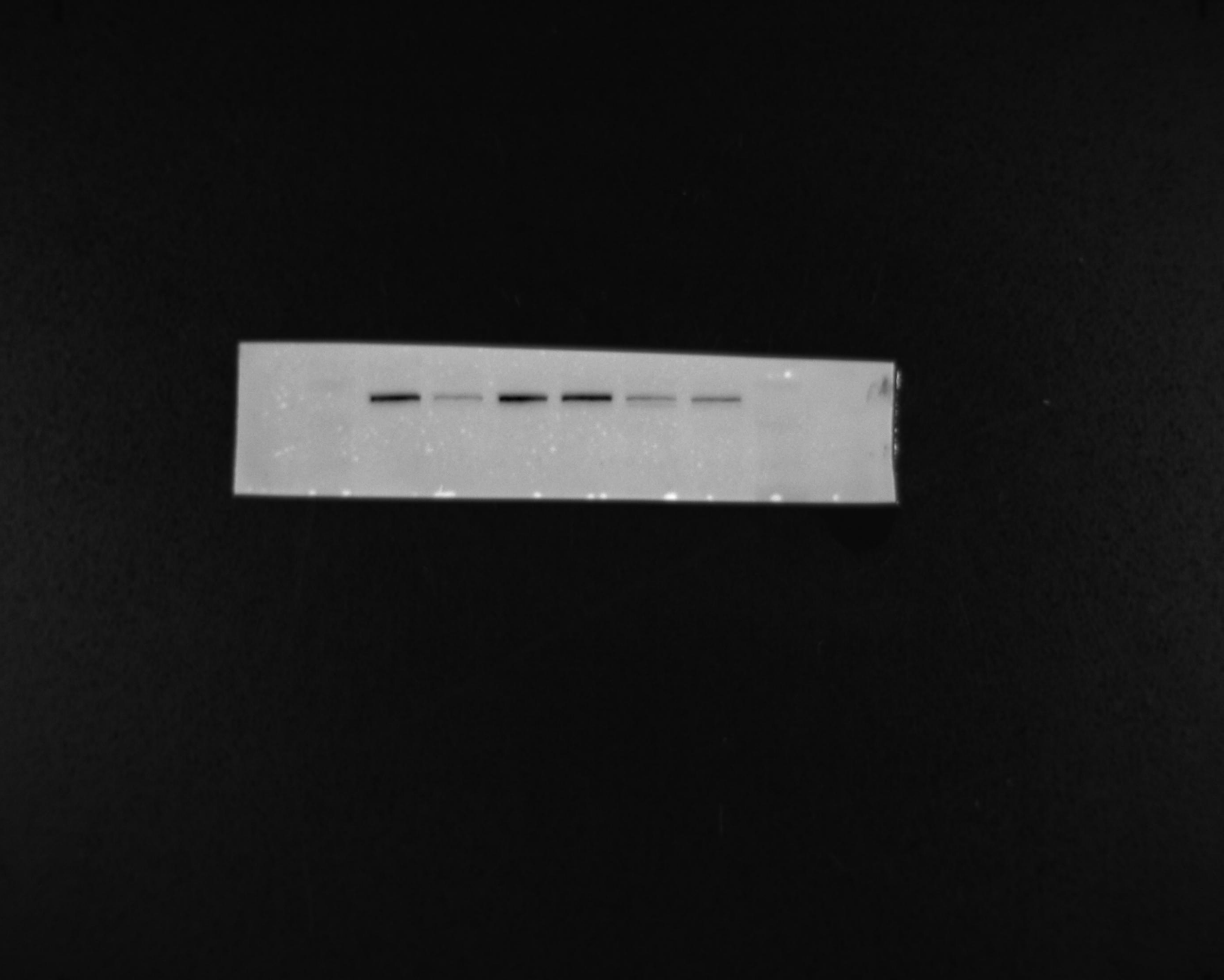

Supplement: Supplementary file 11 [file Data_Sheet_6.ZIP › mGluR5/mGluR5 1-3.tif]

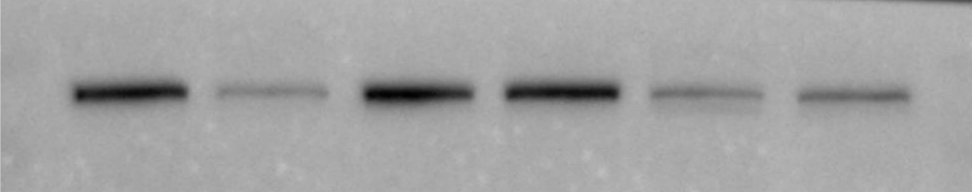

Supplement: Supplementary file 11 [file Data_Sheet_6.ZIP › mGluR5/WPS图片编辑.png]

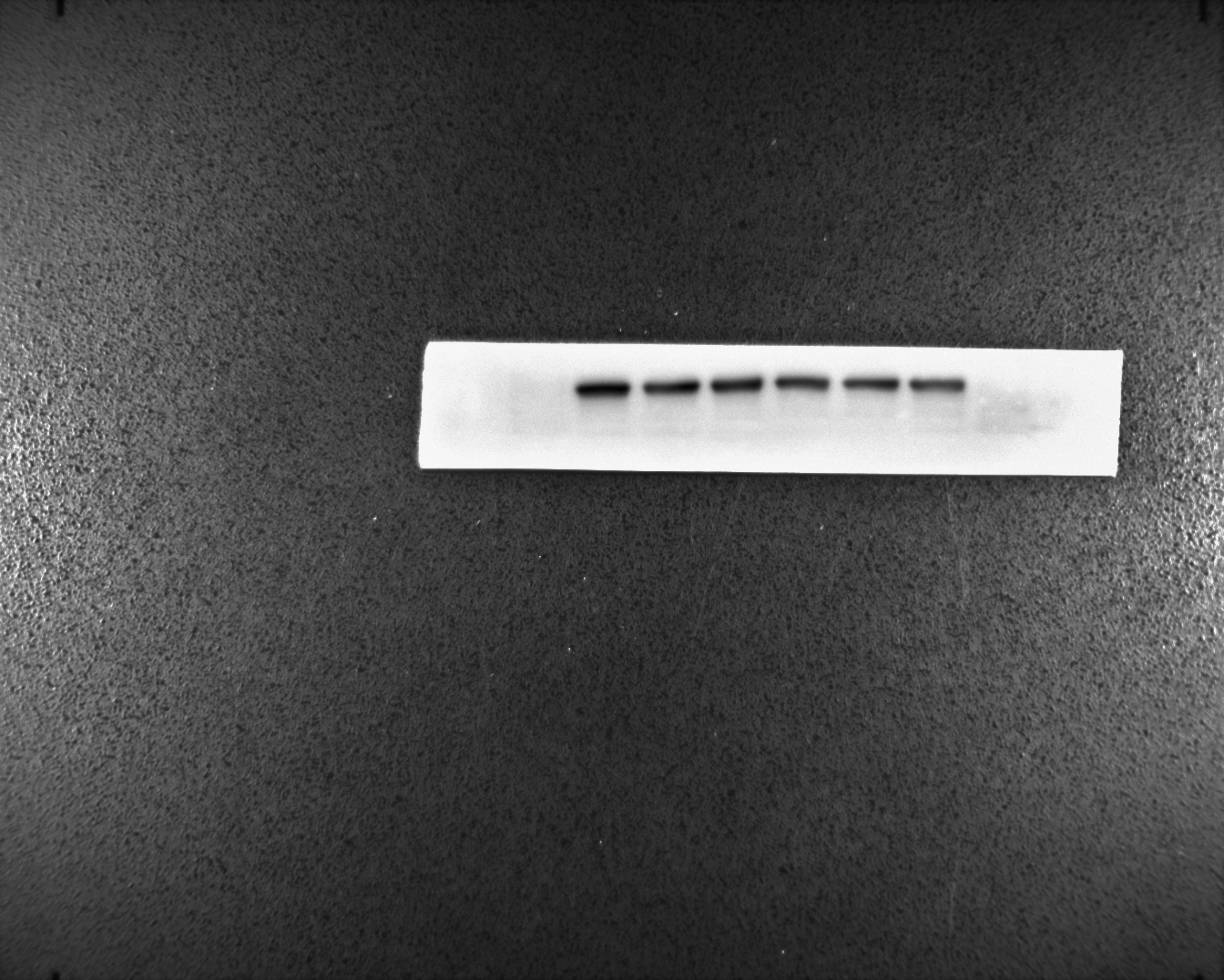

Supplement: Supplementary file 12 [file Data_Sheet_7.ZIP › GAPDH/1.png]

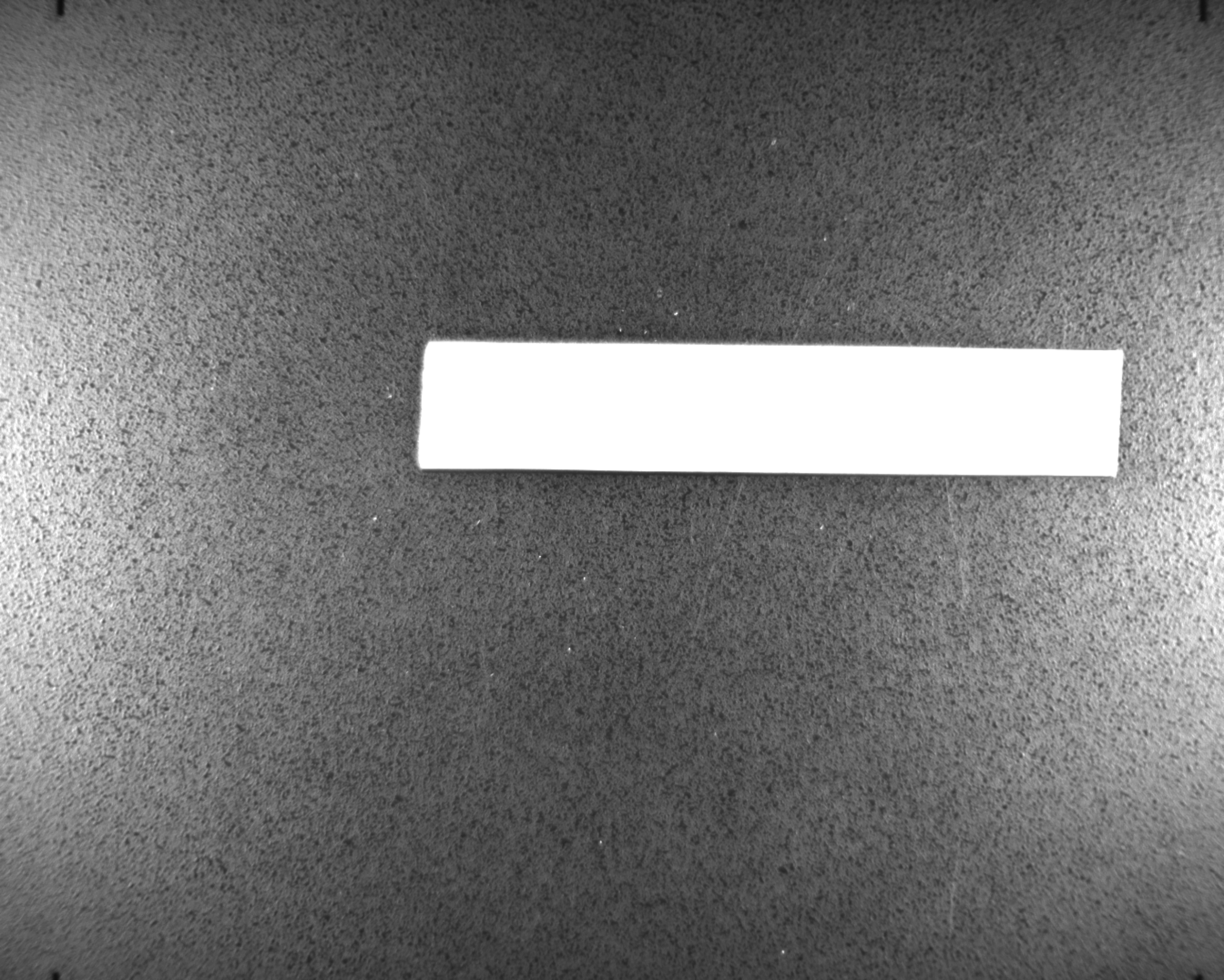

Supplement: Supplementary file 12 [file Data_Sheet_7.ZIP › GAPDH/2.tif]

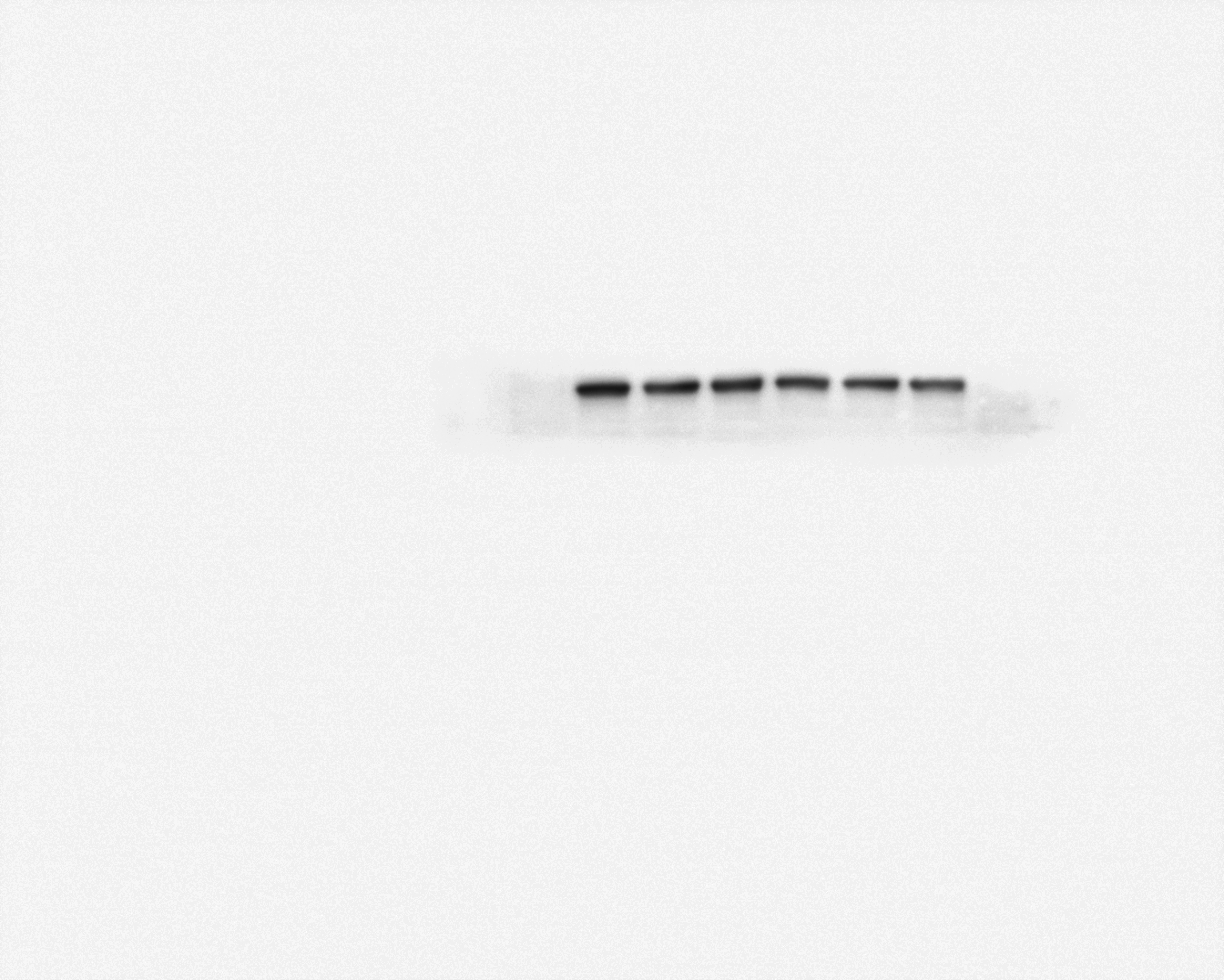

Supplement: Supplementary file 12 [file Data_Sheet_7.ZIP › GAPDH/3.png]

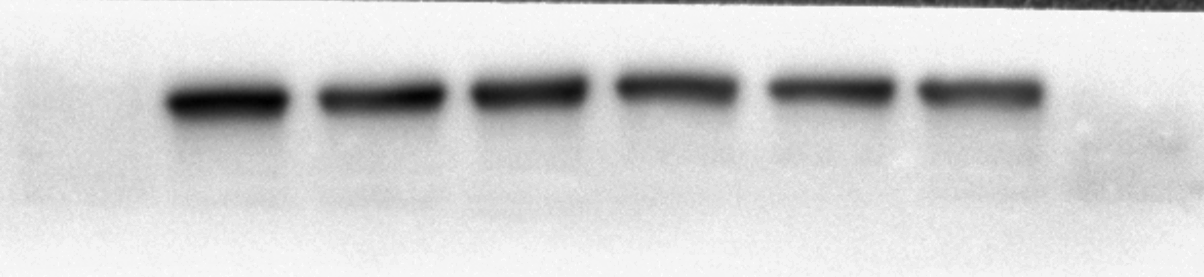

Supplement: Supplementary file 12 [file Data_Sheet_7.ZIP › GAPDH/4.png]

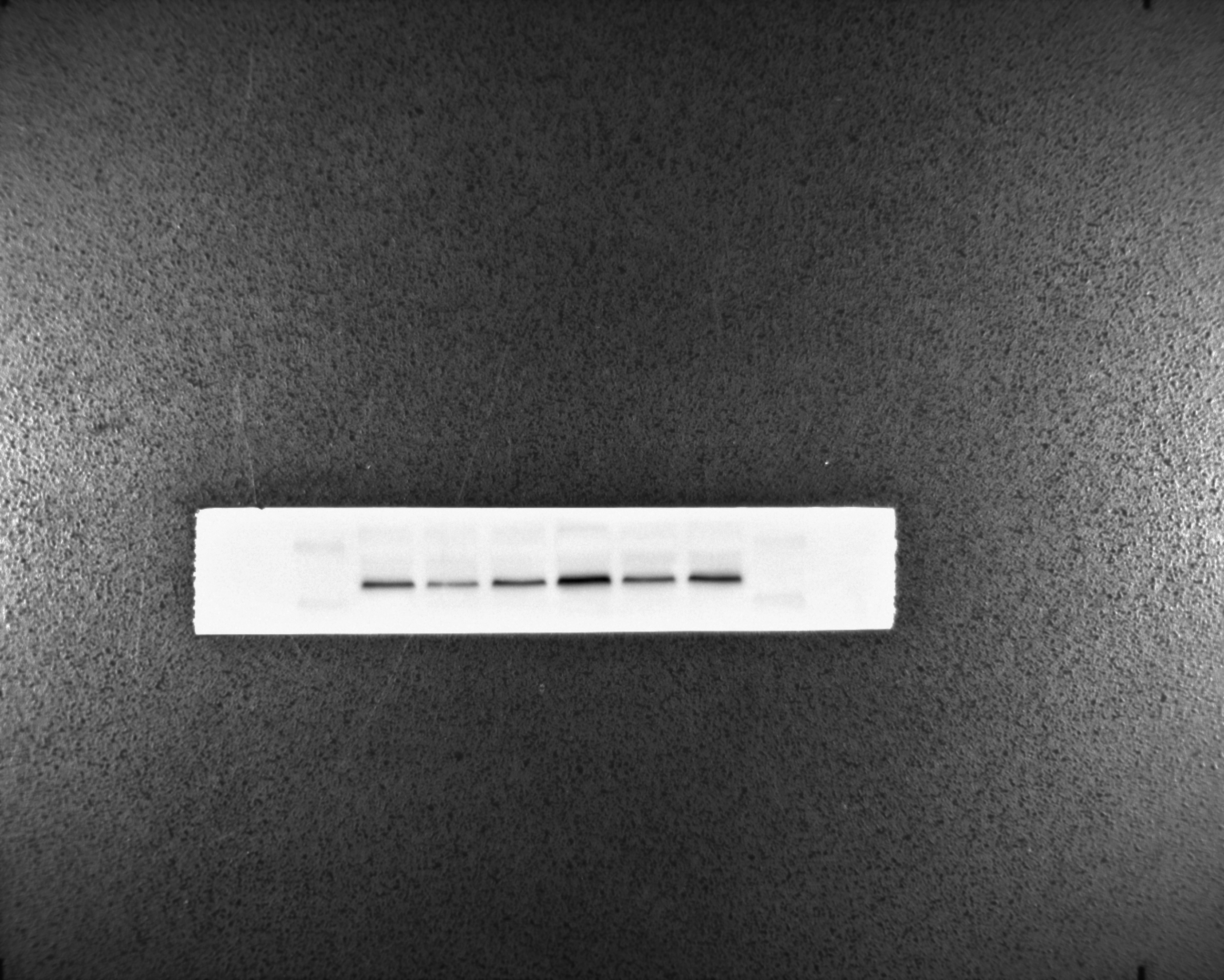

Supplement: Supplementary file 12 [file Data_Sheet_7.ZIP › TH/TH 1-1.png]

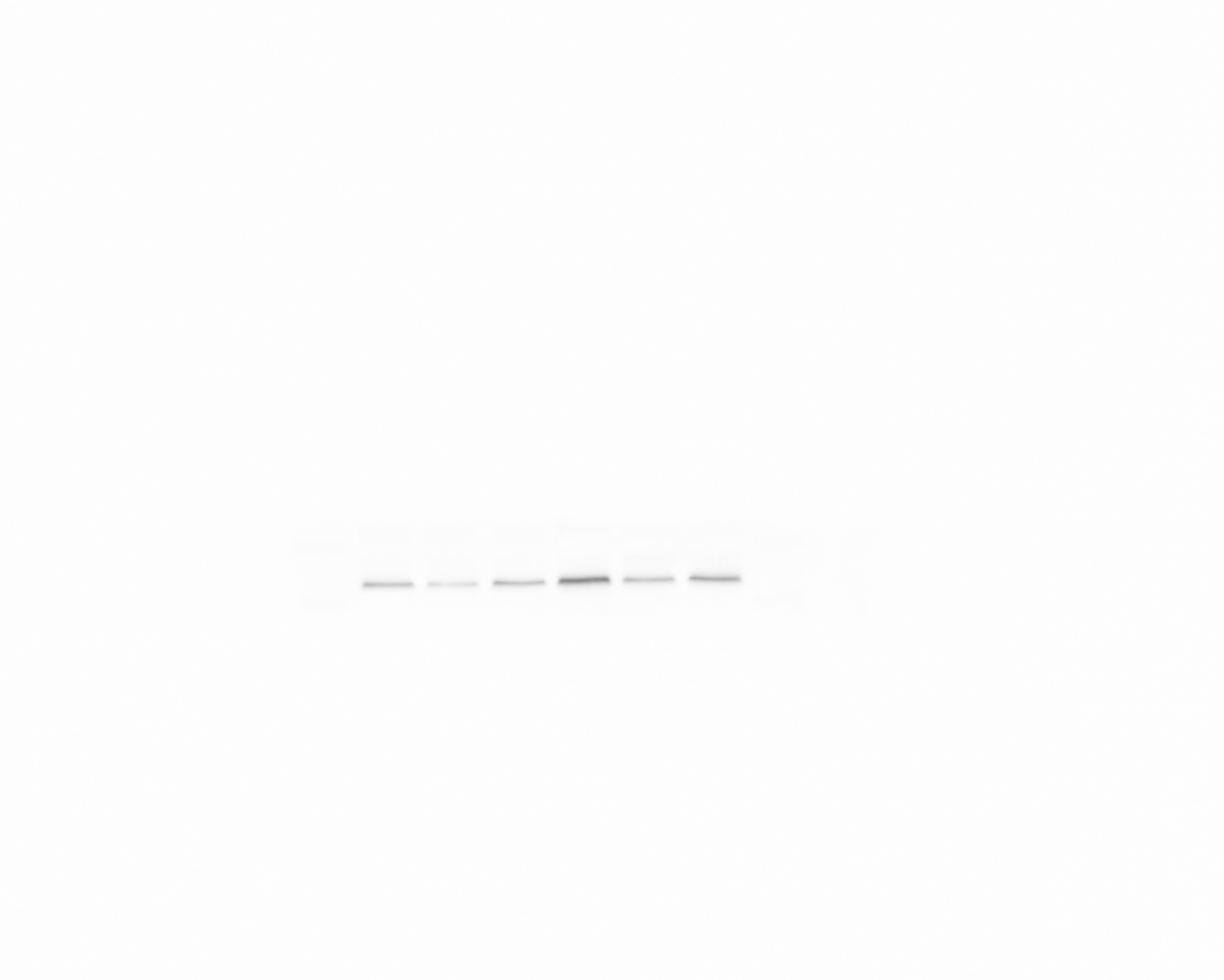

Supplement: Supplementary file 12 [file Data_Sheet_7.ZIP › TH/TH 1-2.png]

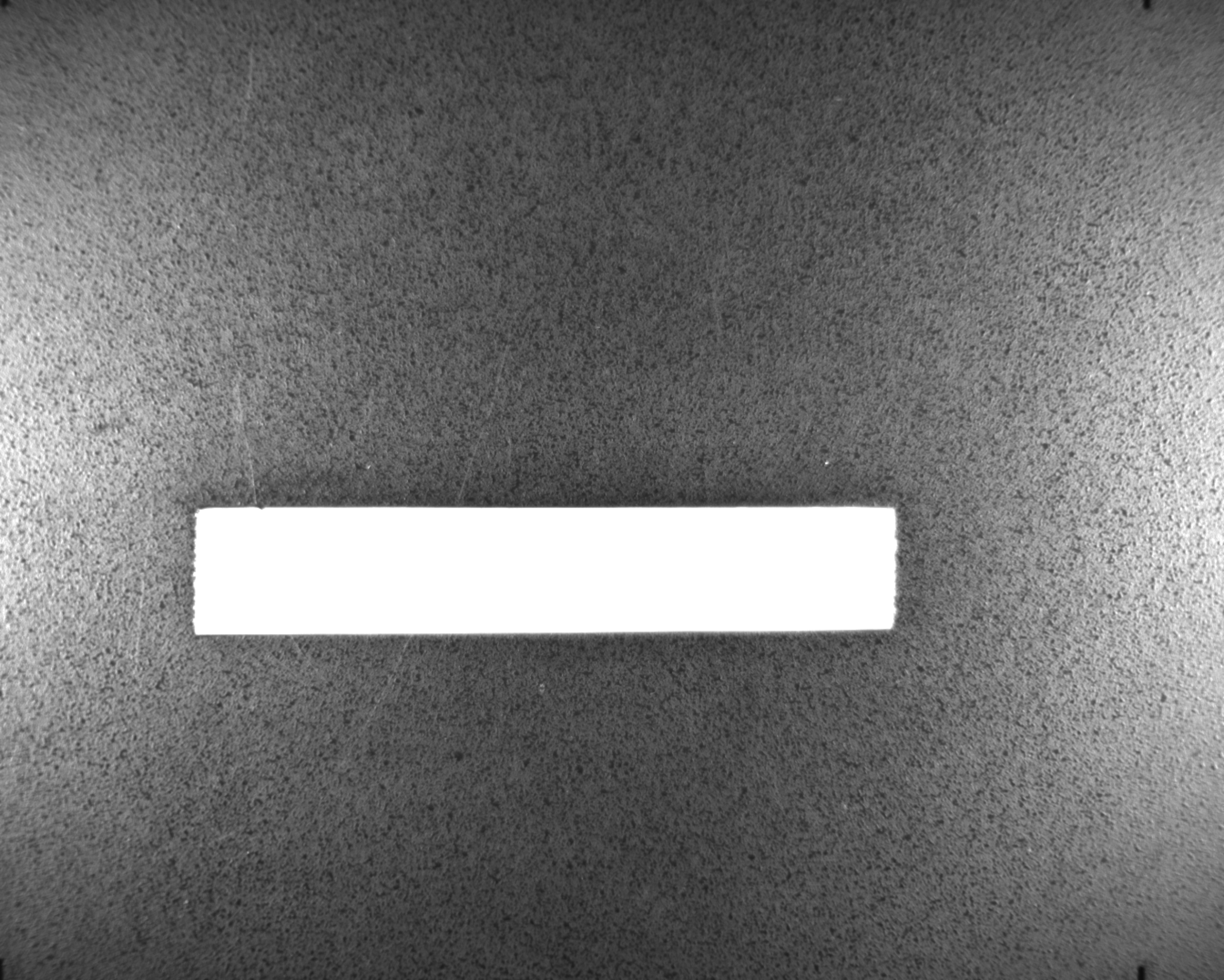

Supplement: Supplementary file 12 [file Data_Sheet_7.ZIP › TH/TH.tif]
